# Insula sub-regions across the psychosis spectrum: morphology and clinical correlates

**DOI:** 10.1038/s41398-021-01461-0

**Published:** 2021-06-04

**Authors:** Julia M. Sheffield, Anna S. Huang, Baxter P. Rogers, Jennifer Urbano Blackford, Stephan Heckers, Neil D. Woodward

**Affiliations:** 1 Department of Psychiatry and Behavioral Sciences, Vanderbilt University Medical Center, Nashville, TN USA; 2 Department of Radiology and Radiological Sciences, Vanderbilt University Medical Center, Nashville, TN USA; 3 Institute of Imaging Science, Vanderbilt University, Nashville, TN USA; 4 Munroe-Meyer Institute, University of Nebraska Medical Center, Omaha, NE USA

**Keywords:** Schizophrenia, Diagnostic markers, Human behaviour

## Abstract

The insula is a heterogeneous cortical region, comprised of three cytoarchitecturally distinct sub-regions (agranular, dysgranular, and granular), which traverse the anterior-posterior axis and are differentially involved in affective, cognitive, and somatosensory processing. Smaller insula volume is consistently reported in psychosis-spectrum disorders and is hypothesized to result, in part, from abnormal neurodevelopment. To better understand the regional and diagnostic specificity of insula abnormalities in psychosis, their developmental etiology, and clinical correlates, we characterized insula volume and morphology in a large group of adults with a psychotic disorder (schizophrenia spectrum, psychotic bipolar disorder) and a community-ascertained cohort of psychosis-spectrum youth (age 8–21). Insula volume and morphology (cortical thickness, gyrification, sulcal depth) were quantified from T1-weighted structural brain images using the Computational Anatomy Toolbox (CAT12). Healthy adults (*n* = 196), people with a psychotic disorder (*n* = 303), and 1368 individuals from the Philadelphia Neurodevelopmental Cohort (PNC) (381 typically developing (TD), 381 psychosis-spectrum (PS) youth, 606 youth with other psychopathology (OP)), were investigated. Insula volume was significantly reduced in adults with psychotic disorders and psychosis-spectrum youth, following an anterior-posterior gradient across granular sub-regions. Morphological abnormalities were limited to lower gyrification in psychotic disorders, which was specific to schizophrenia and associated with cognitive ability. Insula volume and thickness were associated with cognition, and positive and negative symptoms of psychosis. We conclude that smaller insula volume follows an anterior-posterior gradient in psychosis and confers a broad risk for psychosis-spectrum disorders. Reduced gyrification is specific to schizophrenia and may reflect altered prenatal development that contributes to cognitive impairment.

## Introduction

The insula is a heterogeneous brain structure located deep in the Sylvian fissure and implicated in a vast array of human behaviors and mental experiences. Often considered the fifth cerebral lobe, the insula is the first cortical region to develop and differentiate, starting at just six weeks of fetal life^1^. Due to its diverse role in a variety of cognitive, affective, and regulatory functions, the insula is increasingly implicated in psychiatric disorders, with particular interest in its role in psychosis^2^. Insula abnormalities are ubiquitous in psychotic disorders and include structural changes^3^, abnormal resting-state functional connectivity^4,5^, and altered task-based activation^6,7^. Of these, volume deficits are among the most commonly reported.

Despite apparent insula abnormalities in psychotic disorders, several questions remain. First, the anatomical specificity of these abnormalities remains unclear. The insula is comprised of three granular sub-regions (agranular, dysgranular, and granular) distinguished by their cytoarchitectonic features, namely the presence/absence of granular layer IV^8,9^. Yet, most studies in psychosis examine the whole insula^10–12^ or report relatively greater deficits in the anterior portion^13,14^. It is therefore unknown whether granular sub-regions demonstrate distinct patterns of structural alterations in psychosis. Resolving this issue is important because, although strongly interconnected along the dorso-ventral and rostro-caudal axes, these sub-regions support distinct functions across affective (agranular), cognitive (dysgranular), and somatosensory (granular) systems^15^. Therefore, investigating sub-regions can help identify whether specific granular regions are more/less affected in psychosis, with implications for understanding structural changes underlying clinical phenotypes.

Relatedly, the diagnostic specificity of insula abnormalities is also unclear. Evidence of volume reductions in schizophrenia is fairly robust^14^ and some studies find abnormalities in bipolar disorder, suggesting that insula dysfunction is transdiagnostic^16^. However, findings in bipolar disorder are mixed, possibly due to limited number of studies, small sample sizes, and heterogeneous bipolar cohorts comprised of those with and without psychotic features^17–19^.

In addition, the onset and trajectory of insula abnormalities are uncertain. Two distinct approaches can be taken to address this question. First, examination of insula structure in high-risk or prodromal individuals as well as in adults with psychotic disorders ranging from the first episode to chronic, can identify the presence of structural alterations at different stages of illness progression. Prior volumetric studies in prodromal and high-risk youth support this developmental basis^20,21^. In high-risk youth, conversion to psychosis has been associated with smaller insula volume at baseline and a greater rate of insula volume loss over four years^20^. Smaller insula volume is also observed in patients experiencing their first psychotic episode^18,22^. Meta-analyses of the whole brain identify smaller insula volume in high-risk, first episode, and chronic psychosis compared to controls^23,24^, suggesting that insula volume changes observed in chronic schizophrenia patients are present early on in the disease course. Yet, a comprehensive analysis of insula sub-regional structure at different illness stages has not before been investigated.

Second, the developmental window of alteration is unknown; characterization of different morphological features of the insula may point to perturbation of specific developmental stages, including prenatal brain development. The majority of prior work on insula structure in psychosis has focused on volume, which is at its highest in childhood and declines throughout the lifespan^25,26^; however, parallel examination of multiple structural measures may yield additional insights in the timing and etiology of insula abnormalities. Intriguingly, aspects of structural maturation, including thickness, gyrification, and sulcal depth, appear to be driven by different biological processes^27–30^ with distinct developmental trajectories^31^ and genetic influences^32^, suggesting that insult at different developmental stages could differentially impact structural metrics. Gyrification, for instance, develops prenatally (primarily third trimester)^33^ with relatively little change during adolescence^34^. Sulcal depth increases considerably from birth to two years^35^, followed by relative stability or subtle decrease by adolescence^36,37^. Cortical thickness generally increases over years one to six, in line with ongoing development of dendrites and glial cells in childhood, then begins to decrease by age eight in the context of synaptic pruning^37,38^.

While relatively understudied compared to volume, cortical thinning^39–41^, abnormal gyrification^40,42^, and reduced sulcal depth^43^ of the insula have been observed in small samples of psychotic disorder participants using whole brain approaches. Few studies, however, have characterized these structural features in the same cohort, limiting conclusions about the specificity of structural alterations in psychosis. Furthermore, whether morphometric measures of the insula (e.g., thickness, gyrification, and sulcal depth) are altered in psychosis-spectrum youth has never before been examined. Therefore, in addition to providing a detailed account of insula structure across the psychosis spectrum, investigation of different structural metrics has implications for understanding the developmental etiology of insula alterations.

Finally, elucidating insula abnormalities across the psychosis spectrum is clinically relevant to psychosis phenotypes^2,11,44^. As previously noted, insula sub-regions are distinctly involved in affective, cognitive, and somatosensory processing^15,45^, suggesting that sub-regional alterations may differentially contribute to psychotic symptoms^46^. Each sub-region has distinct afferent and efferent connections^47^, recapitulated in their functional and structural connectivity profiles^48,49^. Studies examining insula functioning have described large anterior and posterior clusters^45^ that can be further sub-divided based on their involvement in distinct behavioral domains^50^. Most anterior, the agranular insula has reciprocal connections with limbic regions and is implicated in emotion processing and social cognition^50^. We, therefore, hypothesized it would contribute to negative symptoms, as recently demonstrated using functional connectivity^4^. The middle dysgranular sub-region receives projections from the cingulate cortex and striatum and projects to parietal and prefrontal cortices, supporting its involvement in higher-order cognition (e.g., memory, attention)^4,51^. Relationships with overall cognitive ability were therefore investigated. Finally, the most posterior granular sub-region is considered a “primary interoceptive cortex” with somatotopic representations of objective physiological changes^47^ and involvement in perception. We, therefore, expected structural abnormalities of this region to show relationships with positive symptoms.

The current study quantified volume and morphological characteristics of insula sub-regions in two independent cohorts: adults with psychotic disorders and a community-ascertained developmental cohort^52^ that includes youth with psychosis-spectrum symptoms. We addressed the following aims: (1) characterize volume and surface-based morphology of insula sub-regions across the psychosis spectrum, (2) determine diagnostic specificity of these alterations, and (3) examine contributions to clinical phenotypes.

## Methods and materials

### Participants

Two independent cohorts were used to examine insula structure in adults with a psychotic disorder (Psychosis Cohort) and psychosis-spectrum youth (PNC).

*Psychosis Cohort:* The Psychosis Cohort came from a repository study of 593 individuals that participated in one of three neuroimaging projects conducted in the Department of Psychiatry and Behavioral Sciences at Vanderbilt University Medical Center (VUMC). After excluding individuals that did not meet our study criteria, including neuroimaging quality assurance, our final sample (Table 1) included 196 healthy individuals and 303 individuals with a primary psychotic disorder (schizophrenia spectrum = 211; bipolar disorder with psychotic features = 92). Psychosis symptoms and cognitive ability were measured using the Positive and Negative Syndrome Scale (PANSS)^53^ and the Screen for Cognitive Impairment in Psychiatry (SCIP)^54^, respectively. The SCIP includes measures of verbal memory (immediate and delayed), working memory, verbal fluency, and processing speed and has been shown to be a reliable and valid measure of cognitive ability in psychotic disorders^55^. SCIP subtest raw scores were converted to z-scores using normative data and averaged to create a composite z-score^54^. Symptom severity was assessed using the PANSS, which rates positive, negative, and general psychopathology symptoms over the past two weeks^56^. Average positive and negative scale scores were used to measure positive and negative symptom severity.

**Table 1:** Demographics.

	Healthy participants *N* = 196	Psychosis participants *N* = 303	Statistic		
Age	28.70 (10.20)	29.33 (11.21)	t(497) = 0.63, *p* = 0.527		
Gender (M/F)	120/76	196/107	Χ^2^ = 0.61, *p* = 0.433		
Race (W/AA/O)	139/48/10	220/67/16	Χ^2^ = 6.89, *p* = 0.229		
Education*	15.24 (2.10)	13.53 (2.22)	t(469) = 8.31, *p* < 0.001		
Parental education	14.70 (3.09)	14.81 (3.40)	t(449) = 0.33, *p* = 0.739		
Cognitive functioning					
Premorbid IQ	111.22 (11.00)	102.22 (15.24)	t(483) = 7.06, *p* < 0.001		
Cognitive ability	0.11 (0.62)	−0.93 (0.97)	t(484) = 13.28, *p* < 0.001		
Clinical characteristics					
Positive symptoms	—	16.82 (8.23)	—		
Negative symptoms	—	14.23 (6.62)	—		
Duration of illness (years)		7.72 (10.3)			
CPZ-Equivalent	—	317.07 (211.11)	—		

	Typically Developing *N* = 381	Psychosis Spectrum *N* = 381	Other Psychopathology *N* = 606	Statistics	Comparison
Age	14.06 (3.69)	15.93 (3.05)	14.73 (3.64)	F(2,1365) = 28.03, *p* < 0.001	TD < OP < PS
Gender (M/F)	194/187	175/206	274/332	Χ^2^ = 3.30, *p* = 0.192	
Race (W/AA/O)	209/127/45	116/218/47	292/246/68	Χ^2^ = 54.58, *p* < 0.001	
Education	7.03 (3.62)	8.22 (2.71)	7.67 (3.37)	F(2,1365) = 12.53, *p* < 0.001	TD < OP < PS
Parental Education	14.47 (2.45)	13.45 (2.15)	14.13 (2.21)	F(2,1365) = 12.53, *p* < 0.001	PS < OP < TD
Cognitive functioning					
Premorbid IQ	105.71 (15.77)	98.11 (16.64)	102.52 (15.90)	F(2,1363) = 21.56, *p* < 0.001	PS < OP < TD
Cognitive ability	0.03 (0.55)	0.001 (0.54)	0.06 (0.55)	F(2,1365) = 1.41, *p* = 0.24	
Clinical characteristics					
Positive symptoms	2.07 (4.26)	22.39 (14.03)	4.13 (6.14)	F(2,1364) = 658.13, *p* < 0.001	TD < OP < PS
Negative symptoms	0.25 (0.80)	2.17 (2.87)	0.48 (1.03)	F(2,1365) = 150.0, *p* < 0.001	TD < OP < PS
Duration of illness (years)	—	—	—	—	—
CPZ-Equivalent	—	—	—	—	—

Premorbid IQ for by the Wechsler Test of Adult Reading (WTAR) in the psychosis cohort and the Wide-Ranging Achievement Test (WRAT-4) in the PNC. Cognitive Ability measured by the Screen for Cognitive Impairment in Psychiatry (SCIP) in the Psychosis Cohort and the Penn Computerized Neurocognitive Battery (CNB) in the PNC. Positive and negative symptoms measured by the Positive and Negative Syndrome Scale (PANSS) in the psychosis cohort. In the PNC they were measured by the PRIME screen (positive symptoms) and the Structured Interview for Prodromal Symptoms (SIPS) (negative symptoms). *AA* African American, *CPZ* Chlorpromazine, *F* Female, *M* Male, *O* Other, *PANSS* Positive and Negative Syndrome Scale, *W* White.

*Philadelphia Neurodevelopmental Cohort (PNC):* The PNC was obtained from the database of Genotypes and Phenotypes (dbGaP: Study Accession phs00607.v3.p2), and consists of 9498 youth aged 8–21. Of the 1601 participants that completed a neuroimaging session^57^, 1368 were included in the current study (Table 1) after excluding participants that did not meet our inclusion criteria, including neuroimaging quality assurance. Participants were classified as typically developing (TD) (*n* = 381), psychosis-spectrum (PS) (*n* = 381), or other psychopathology (OP) (*n* = 606) using similar procedures as prior PNC studies^58^. OP participants had suprathreshold psychopathology symptoms but did not meet PS criteria. Positive and negative symptoms were assessed using the PRIME Screen-Revised and Scale for Prodromal Syndromes^52^. Cognition was assessed using the Penn Computerized Neurocognitive Battery (CNB), which includes measures of executive-control, episodic memory, complex cognition, and social cognition^59^. Overall cognitive ability (average z-score of accuracy on each cognitive test) was used as a measure of cognition. Details on study procedures and group assignment are included in the Supplement.

All four studies (three neuroimaging projects at VUMC and the PNC study) were approved by the Institutional Review Board (IRB) at each site and all participants provided signed written informed consent prior to completing study procedures.

### Neuroimaging data acquisition

Imaging data for the Psychosis Cohort were collected on one of two identical 3.0-T Philips Intera Achieva MRI scanners (Philips Healthcare, Andover, MA), located at the Vanderbilt University Institute of Imaging Science. A T1-weighted anatomical image was collected on all subjects and visually inspected for quality, blinded to diagnosis. The Psychosis Cohort was collected across three neuroimaging studies with slightly different scanning parameters (e.g., minor differences in TR/TE), and somewhat different inclusion criteria (e.g., one study emphasized recruitment of early psychosis patients while another included a large proportion of the bipolar participants). Therefore ‘study’ was included as an additional covariate in all neuroimaging analyses of the Psychosis Cohort. Imaging data for the PNC was collected on a Siemens TIM Trio scanner located at the Hospital of the University of Pennsylvania. A T1-weighted scan was obtained using a magnetization-prepared rapid acquisition gradient echo (MPRAGE) sequence. Additional information on neuroimaging data acquisition and quality assurance are available in the Supplement.

### Insula volume and surface features

Brain volumes and cortical surface features (i.e., thickness, gyrification, sulcal depth) were quantified using voxel-based morphometry (VBM) and surface-based morphometry (SBM), respectively, as implemented in the Computational Anatomy Toolbox 12 (CAT12) for Statistical Parametric Mapping version 12 (SPM12)^60^.

*Voxel-Based Morphometry*: T1 images were pre-processed in CAT12 using recommended processing procedures which include: correction for bias-field inhomogeneities, spatial registration to a reference brain using linear (12-parameter affine) and non-linear transformations, segmentation into gray matter, white matter, and cerebrospinal fluid tissue classes, and spatial normalization to MNI-space using the DARTEL algorithm. The normalized, modulated gray matter segmentations were used in the statistical analyses described below.

*Surface-Based Morphometry*: The CAT12 toolbox uses the projection-based thickness (PBT) method to automatically reconstruct the cortical surface^61^. Following surface reconstruction, morphometric indices were calculated, including cortical thickness, gyrification, and sulcal depth. These metrics provide complimentary and non-overlapping features of insula structure. Cortical thickness estimates the thickness of the gray matter surface using segmentation of the distance between inner and outer surfaces of white and gray matter boundaries^62^. Gyrification was estimated using a curvature-based approach to measure absolute mean curvature, with greater cortical folding contributing to greater local gyrification, as described by Luders and colleagues^63^. Sulcal depth was estimated based on the Euclidean distance between the central surface and its convex hull, using square-root transformed values. As recommended by the developers, cortical thickness data was resampled and smoothed at 15 mm FWHM, whereas gyrification and sulcal depth data were resampled and smoothed at 20 mm FWHM. The distance between sulci and gyri is approximately 20–30 mm, so a 20 mm smoothing kernel enhances the features within this range^63^.

### Neuroimaging statistical analysis

Volume and surface features of the insula were analyzed using complimentary region-of-interest (ROI) and voxel/vertex-wise approaches.

*ROI Analysis:* A-priori masks of agranular, dysgranular, and granular sub-regions, as determined by Farb and colleagues^64^, were used to extract insula sub-region volumes from each individuals normalized, modulated gray matter segmentation^65^. The Farb atlas was chosen due to its use of well-characterized cytoarchitectonic divisions^9^ to identify granular sub-regions. This allowed, not only for the examination of structurally defined sub-regions, but also limited the use of maps based on functional activation or connectivity, which could be less reliable in schizophrenia^5^. The HCP-MMP1 atlas^66^, a surface-based atlas which includes insula sub-regions was used to extract average thickness, gyrification, and sulcal depth from the surface meshes for insula sub-regions. Volume and surface features were analyzed separately in SPSS v.26. Repeated-measures ANCOVA was conducted in each cohort with insula sub-region as a repeated factor and diagnostic group as a between-groups factor. Age, gender, total intracranial volume (TIV), study (for the psychosis cohort only), and topography defects (SBM analyses only) were included as covariates. Significant main effects of the group were followed-up with analysis of group differences for each sub-region using multivariate ANCOVA. Significance was Bonferroni-corrected for three sub-regions (critical alpha = 0.017). Within the Psychosis Cohort, effects of diagnosis (schizophrenia-spectrum and bipolar disorder) and illness stage (early and chronic psychosis) were also examined using repeated-measures ANCOVA as described above. Finally, group differences in gyrification and sulcal depth using the sub-region approach were supplemented by examination of the whole insula using the Desikan-Killiany (DK40) atlas parcellation^67^. This was done to ensure sufficient area to measure cortical folding, in order to help validate sub-regional analyses.

*Voxel/vertex-wise analyses*: Voxel-wise analysis of insula volume was performed in SPM12 by entering the modulated gray matter images (smoothed with a 4 mm kernel) into an ANCOVA, masked to include only voxels in the a-priori defined whole insula mask^64^. Similarly, vertex-based analysis of cortical thickness, gyrification, and sulcal depth was analyzed using one-way ANCOVAs for each surface metric within CAT12, using the HCP-MMP1 whole insula as an explicit mask. Age, gender, TIV, study (Psychosis cohort only), and topological defects (SBM analysis only) were included as covariates. The resultant statistical parametric maps were thresholded at cluster-level corrected *p*_FWE_ = 0.05 for voxel-wise *p* < 0.001 uncorrected.

### Associations with cognitive and clinical phenotypes

In the psychosis cohort, relationships between structure and cognition were assessed in the whole sample controlling for the group (control, schizophrenia, and bipolar), whereas relationships with symptoms were assessed in the psychosis patients only. In the PNC, all correlations were conducted across the entire sample, controlling for group. A-prior associations were first tested: agranular insula and negative symptom severity, dysgranular insula and cognitive ability, granular insula and positive symptom severity. To examine specificity, two-tailed a-priori correlations were followed-up with examination of all pairwise brain-behavior relationships. Associations were tested using partial correlation with group, age, gender, TIV, and topography defects (for SBM measures) as covariates. A-priori correlations were Bonferroni-corrected for four metrics (critical alpha = 0.013). All other correlations are Bonferroni-corrected for the additional 8 tests (critical alpha = 0.006).

## Results

### Group differences

Structural differences across sub-regions (main effect of sub-region) are presented in Table S1.

#### Psychosis cohort

**Fig. 1:**
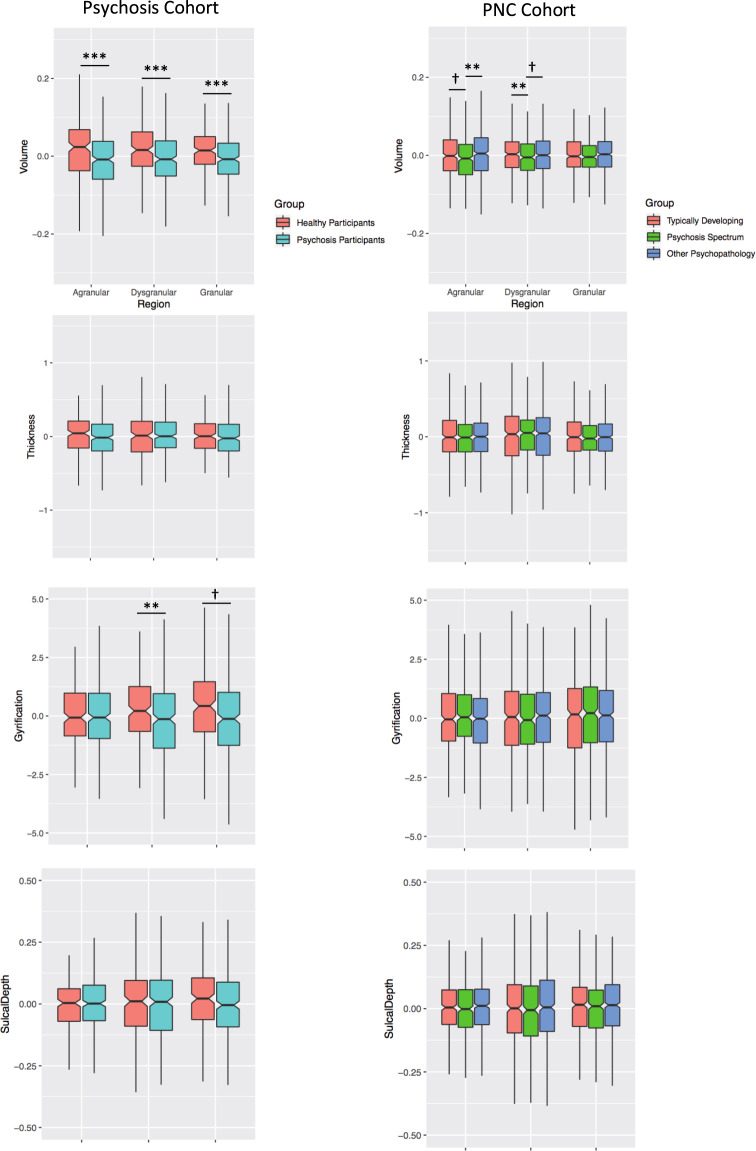
Insula volume and surface-features based on region-of-interest analyses. Insula volume was extracted from granular sub-regions in all participants using masks from Farb and colleagues^64^. Psychotic disorder participants and psychosis-spectrum youth exhibited reduced volume of insula sub-regions, following an anterior-posterior gradient. Surface-based morphometry values were extracted from granular sub-regions based on parcellations from the HCP-MMPI atlas^66^. Significant alterations were observed only for dysgranular gyrification within the psychotic disorders cohort. Marginal means are presented. Covariates included age, gender, total intracranial volume (TIV), study (for psychotic disorders cohort), and topography defects (for surface-based measures). ***p < 0.001, **p < 0.017, ^†^*p* < 0.05 but did not survive multiple comparisons correction.

**Fig. 2:**
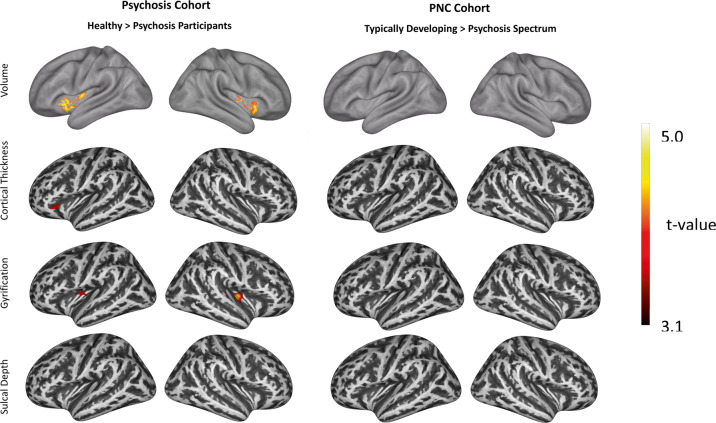
Insula volume and surface-features based on voxel-wise and vertex-based analyses. To complement our region-of-interest analyses, we conducted voxel-wise (volume) and vertex-based (surface) analyses of insula structure in each cohort using explicit insula masks^64,66^. Group differences within the psychosis (healthy controls > psychosis) and PNC (typically developing > psychosis spectrum youth) cohorts are displayed. Mirroring and extending our region-of-interest analyses, psychotic disorder participants had reduced volume and gyrification and well as reduced cortical thickness. No significant group differences were observed in the PNC. Cluster-level corrected *p*_FWE_ = 0.05 for voxel-wise *p* < 0.001 uncorrected, controlling for age, gender, total intracranial volume (TIV), study (for psychotic disorders cohort), and topography defects (for surface-based measures).

*Volume:* In the ROI-analysis, we observed a significant main effect of group (F(1,493) = 16.94, *p* < 0.001; 4.28% smaller) and group by sub-region interaction (F(2,986) = 3.66, *p* = 0.03) (Fig. 1). Follow-up analyses indicated that while all insula sub-regions were smaller in psychosis (all *p*-values ≤ 0.001), there was an anterior–posterior gradient. Specifically, reduced insula volume compared to healthy controls was most pronounced in the agranular (5.22% smaller), then dysgranular (4.48% smaller) then granular sub-regions (3.56% smaller). These findings were recapitulated in our voxel-wise analysis, which revealed large bilateral clusters of reduced insula volume in psychosis spanning all sub-regions (Fig. 2; Table S2).

*Cortical Thickness*: There was no significant main effect of group (F(1,492) = 0.40, *p* = 0.53) or group by sub-region interaction (F(2,984) = 2.37, *p* = 0.09) in the ROI-analysis. Vertex-based analysis revealed a cluster in the agranular insula [−34 31 −2] with significantly reduced cortical thickness in psychosis (Fig. 2; Table S2).

*Gyrification:* In the ROI-analysis, gyrification was significantly reduced in psychosis (F(1,492) = 6.51, *p* = 0.01) (Fig. 1) with a non-significant group by sub-region interaction (F(2,984) = 2.46, *p* = 0.09). Using the DK40 atlas, whole insula gyrification was still significantly reduced in psychosis (F(1,492) = 8.81, *p* = 0.003). Vertex-based analysis identified two clusters with reduced gyrification in psychosis, with one peak in the right dysgranular insula [36 −21 3] and the other peak in the left granular insula [−35 −9 11] (Fig. 2; Table S2).

*Sulcal Depth:* No differences were detected between psychosis and healthy participants in either the ROI-analysis (F(1,492) = 0.00, *p* = 0.99) or vertex-based analysis (no significant clusters). When using the DK40 atlas, whole insula sulcal depth did not differ between groups (F(1,492) = 0.03, *p* = 0.854). In the sub-regional ROI-approach, there was a significant group by region interaction (F(2,984) = 4.82, *p* = 0.008). Groups did not differ for any insula sub-region (*p*’s > 0.26), however, healthy subjects had relatively greater granular sulcal depth while psychosis participants had relatively smaller granular sulcal depth (Fig. 1). In both groups, all sub-regions significantly differed from one another (*p*’s < 0.001), with granular insula having the greatest sulcal depth and agranular insula having the smallest sulcal depth in both groups.

#### Diagnostic effects (Schizophrenia-spectrum and psychotic bipolar disorder)

Repeated-measured ANCOVA revealed that the structural abnormalities reported in the psychosis sample were specific to schizophrenia and not observed in psychotic bipolar disorder (Full results are presented in Table S3).

Regarding volume, all three sub-regions were smaller in schizophrenia compared to bipolar participants (*p*’s < 0.04). Bipolar participants did not significantly differ from controls in any sub-region.

Analysis of morphometry revealed that lower dysgranular gyrification was also specific to schizophrenia. When compared with controls, schizophrenia (1.87% reduced, *p* = 0.002) but not bipolar (0.72% reduced, *p* = 0.37) participants showed significant reductions. The same was true for DK40 whole insula gyrification, which was significantly reduced in schizophrenia (*p* = 0.001), but not bipolar disorder (*p* = 0.43) (bipolar patients were intermediate to schizophrenia and healthy controls). Cortical thickness did not differ across the three groups (F(2,491) = 2.06, *p* = 0.13). Sulcal depth was slightly increased in bipolar participants as compared with controls (0.71% greater, *p* = 0.03) and schizophrenia participants (1.01% greater, *p* = 0.002), which was true also when considering the DK40 whole insula (bipolar vs. controls: *p* = 0.05; bipolar vs. schizophrenia: *p* = 0.003).

#### PNC cohort

*Volume:* We observed a significant main effect of group (F(2,1362) = 4.23, *p* = 0.015) and a non-significant group by region interaction (F(4,2724) = 1.11, *p* = 0.35). Similar to psychosis patients, PS youth demonstrated an anterior-posterior gradient with the largest volume reduction in the agranular (1.46% smaller), then dysgranular (1.38% smaller), then granular (1.03% smaller) sub-regions when compared with TD youth (Fig. 1). The same pattern was seen when PS youth were compared with OP (agranular: 1.6% smaller; dysgranular: 1.07% smaller; granular: 0.86% smaller). TD and OP youth had similar overall insula volumes (*p* = 0.81). In the voxel-wise analysis, a cluster localized to the agranular sub-region [38 15−10] (33 voxels) was reduced in PS youth; however, it fell short of our conservative correction for multiple comparisons (*p*_FWE_ = 0.06; Supplementary Fig. 1; Table S2).

*Structure-Based Morphometry:* There were no differences between groups in insula cortical thickness (F(2,1361) = 0.04, *p* = 0.97), gyrification (F(2,1361) = 0.29, *p* = 0.75), or sulcal depth (F(2,1361) = 1.48, *p* = 0.23). There were also no significant region by group interactions (thickness: F(4,2722 = 0.32, *p* = 0.87); gyrification: F(4,2722) = 2.03, *p* = 0.09); sulcal depth: F(4,2722) = 1.62, *p* = 0.17)). No group differences were observed for DK40 whole insula gyrification (F(2,1361) = 0.18, *p* = 0.836) or sulcal depth (F(2,1361) = 1.81, *p* = 0.16). Results were similar for the vertex-based analysis (Fig. 2).

### Associations with cognitive and clinical phenotypes

Group differences between the cognitive and clinical phenotypes are presented in Table 1. All brain–behavior relationships are presented in Table S4 and summarized below. Within the psychosis cohort, we also examined group differences between bipolar and schizophrenia-spectrum participants. Schizophrenia participants had significantly worse cognitive ability (F(1, 262) = 9.12, *p* = 0.003), positive (F(1, 265) = 6.70, *p* = 0.01) and negative (F(1, 265) = 36.98, *p* < 0.001) symptoms as compared with psychotic bipolar disorder participants.

#### Agranular structure-negative symptoms

Agranular insula volume (*r* = −0.24, *p* < 0.001) and thickness (*r* = −0.23, *p* < 0.001) were significantly associated with negative symptoms in psychotic disorders, but not in the PNC (*p*’s > 0.05).

#### Dysgranular structure-cognitive ability

**Fig. 3:**
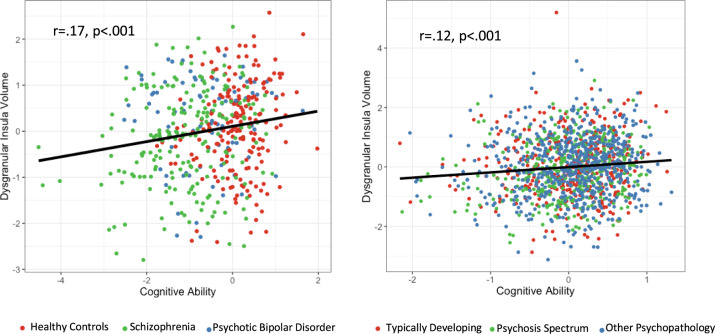
Dysgranular volume and cognition. Correlation between dysgranular volume and cognitive ability in the psychotic disorder and PNC cohorts. Volume is presented as a marginal mean controlling for group, age, gender, and total intracranial volume (TIV). In both groups, lower dysgranular insula volume was associated with worse overall cognitive ability, with highly similar effect sizes.

Overall cognitive ability was significantly associated with volume of the dysgranular insula in both the Psychosis Cohort (*r* = 0.17, *p* < 0.001) and PNC (*r* = 0.12, *p* < 0.001) (Fig. 3). In the Psychosis Cohort, dysgranular thickness (*r* = 0.19, *p* < 0.001) and gyrification (*r* = 0.12, *p* = 0.011) were also associated with cognition. In the PNC, greater dysgranular sulcal depth was associated with better cognitive ability (*r* = 0.13, *p* < 0.001) but this relationship was not seen in the Psychosis Cohort (*r* = 0.014, *p* = 0.76).

#### Granular structure-positive symptoms

Positive symptom severity was associated with granular insula thickness (*r* = −0.19, *p* = 0.001) in psychosis patients, but not in PS youth (*r* = 0.01, *p* = 0.83). No other significant relationships were observed.

#### Sub-region and behavior specificity

In addition to the above-reported relationships, we also found that cognitive ability was associated with agranular volume in both the Psychosis Cohort (*r* = 0.22, *p* < 0.001) and the PNC (*r* = 0.11, *p* < 0.001).

In the Psychosis Cohort only, cognition was also related to agranular thickness (*r* = 0.19, *p* < 0.001), granular volume (*r* = 0.21, *p* < 0.001) and granular thickness (*r* = 0.15, *p* < 0.001) indicating that volume and thickness of the entire insula was associated with cognition. Negative symptoms were also associated with volume of the dysgranular (*r* = −0.18, *p* = 0.002) and granular (*r* = −0.18, *p* = 0.002) sub-regions, indicating relationships between whole insula volume and negative symptoms. Positive symptoms were also associated with agranular thickness (*r* = −0.17, *p* = 0.006).

In the PNC, cognitive ability was related to sulcal depth of the agranular (*r* = 0.15, *p* < 0.001) and granular (*r* = 0.09, *p* < 0.001) sub-regions. Positive symptoms were related to agranular gyrification (*r* = 0.08, *p* = 0.004).

### Supplemental analyses

Supplemental analyses were conducted to examine associations between insula structure, antipsychotic medication, and illness stage in psychosis, as well as interactions with age and sex. These findings are presented in the Supplement (see Supplementary Information, including Figs. S2 and S3 and Tables S5–S8). In brief, both early and chronic patients demonstrated smaller insula volumes and lower gyrification as compared with healthy controls. Volume reductions followed an anterior-posterior gradient in both groups.

Furthermore, age and sex effects were detected and are described in detail in the Supplement. Briefly, in both cohorts, volume and sulcal depth were negatively associated with age for all insula sub-regions. Group by age interactions were non-significant except for a group by age interaction for dysgranular gyrification (*r* = 0.14, *p* = 0.002) in the Psychosis Cohort, driven by a slightly negative relationship in the psychosis patients but a zero-slope relationship in the healthy controls. Sex effects were largely non-significant in both cohorts, except for slightly greater granular gyrification in men within the Psychosis Cohort (*p* = 0.003) and slightly greater dysgranular volume (*p* = 0.007), dysgranular thickness (*p* < 0.001), and agranular gyrification (*p* = 0.001) in boys in the PNC. In addition, within the Psychosis Cohort, chlorpromazine (CPZ)-equivalence values did not significantly correlate with any structure metrics (Table S9) suggesting that group differences were unlikely to be driven by medication use. Finally, to mitigate the potential impact of demographic variables (age, gender, race) on the PNC findings, a matched sample of TD and PS youth were identified (Table S10). Insula volume was lower in the PS youth to a similar degree as was observed in the larger sample, but the findings in this smaller sample were not significant (*p* = 0.22).

## Discussion

We examined insula sub-regional volume, morphology, and clinical correlates in adults with psychotic disorders (schizophrenia-spectrum and psychotic bipolar disorder) and youth with psychosis-spectrum symptoms. We found that insula volume is smaller in psychotic disorder patients and psychosis-spectrum youth, and is associated with cognitive impairment in both cohorts. Investigation of insula sub-regions revealed that smaller insula volume follows an anterior-posterior gradient across the psychosis spectrum, with smaller volume being more pronounced in anterior insula. Parallel analysis of surface-based morphometry identified lower gyrification in psychotic disorders, with limited evidence of reduced cortical thickness or sulcal depth. Volume and gyrification reductions were observed only in schizophrenia, not psychotic bipolar disorder. Gyrification was primarily reduced in the dysgranular sub-region, correlating with cognitive deficits. Finally, positive and negative symptoms were associated with smaller volume and thickness of multiple granular sub-regions in psychotic disorders but not psychosis-spectrum youth. Together, these findings suggest insula volume loss as a neurodevelopmental vulnerability that confers broad risk for psychosis-spectrum disorders and is associated with clinical phenotypes, especially cognitive impairment. Insula morphometry, on the other hand, is more specific to reduced gyrification in schizophrenia, pointing to a possibly early neurodevelopmental abnormality in this population.

While prior work has reported on insula volume loss in psychosis, few studies have considered the heterogeneity of insula structure. Cytoarchitecture of the insula changes along the anterior-posterior axis, transitioning from an agranular (anterior) to a granular (posterior) neocortex with a large intermediate dysgranular region^68^. Integration along this axis is believed to underlie the neurobiology of subjective feelings^69^. Although volumetric studies point to whole insula reductions in psychotic disorders, meta-analysis shows relatively smaller volume in anterior than posterior regions^14^. We extend these findings by demonstrating an anterior-posterior gradient of smaller insula volume in psychotic disorders, with the most pronounced reductions (relative to healthy controls) in the agranular insula, followed by the dysgranular and granular sub-regions. We also demonstrate that this pattern of volume differences is observed in youth with psychosis-spectrum symptoms, early and chronic psychosis. These findings suggest, in a cross-sectional sample, that smaller insula volume has origins in early adolescence that confers risk for psychosis-spectrum disorders. Intriguingly, volume deficits were not observed in youth with other psychopathology (e.g., depression, anxiety disorders) but were instead specific to psychosis-spectrum youth. Prior work has demonstrated smaller insula volume in adults with non-psychotic psychiatric disorders^3^, and insula dysfunction is implicated in various psychiatric conditions^70^. Our findings suggest that, during adolescence, insula volume abnormalities may be specific, or at least most pronounced, in individuals with psychosis-spectrum symptoms.

Insula structural alterations were also more pronounced in schizophrenia-spectrum than psychotic bipolar disorder participants. Prior research on insula pathology in bipolar disorders has been mixed^12,16,17,71^. Unlike prior work in heterogeneous bipolar samples, all bipolar participants in our cohort had experienced psychosis. Therefore, we provide additional evidence that those with schizophrenia-spectrum disorders have notable alterations to insula structure that may be distinct from individuals with affective psychosis. This finding also speaks to the weaker structural differences observed in PS youth, many of whom may never convert to schizophrenia-spectrum disorder or develop bipolar disorder^72^. On-going longitudinal follow-up studies of the PNC will further clarify the specificity of insula abnormalities.

Consistent with previous reports, we also observed reduced insula gyrification in psychotic disorders, particularly schizophrenia^17,73,74^. Follow-up analyses revealed reduced gyrification in both early and chronic psychosis, with the most marked deficits in the dysgranular sub-region. Gyrification develops prenatally^75^ and is relatively stable over childhood and adolescence^76^. In youth at clinical-high risk for psychosis, gyrification has been shown to be stable over one year, suggesting that abnormal gyrification reflects very early developmental changes^77^. Gyrification is particularly intriguing in the context of schizophrenia, as cortical folding is closely linked with the development of neural connectivity patterns^78^. In fact, altered gyrification has been put forth as foundational to the global connectivity abnormalities in schizophrenia thought to sub-serve clinical phenotypes^79^. Recently, we have shown altered functional connectivity of insula sub-regions during resting-state in an overlapping sample of schizophrenia participants^4^. Functional differentiation of insula connectivity in schizophrenia is also altered along the anterior-posterior axis^5^. While speculative, our findings support a neurodevelopmental aberrant connectivity model of schizophrenia^80^ with early, possibly prenatal, origins. Our data further suggests that reduced dysgranular gyrification may contribute to the very early manifestation of cognitive deficits observed in those who develop schizophrenia^81^.

Cortical thickness, on the other hand, was not widely reduced. Cortical thickness captures dendritic arborization, myelination, and pruning^82,83^ which increases during the first two years of life^84^, followed by a decrease^37^, similar to trajectories observed for volume. Prior work in schizophrenia and bipolar disorder has shown cortical thinning across many regions of the brain, particularly in schizophrenia, including in the insula^17^. Our vertex-based approach revealed a cluster in the agranular insula with cortical thinning in psychosis, but this was not observed in PS youth. Discrepancies between the widespread volume deficits and relatively small changes in cortical thickness could be explained (at least in part) by differences in surface area. Both thickness and surface area contribute to estimates of volume but are themselves independent^85^. Surface area is highly correlated with our gyrification index^63^, and surface area of the insula has previously been shown to be reduced in schizophrenia^86^.

Insula sulcal depth, which reflects cortical folding related to both early radial (vertical) and later tangential (horizontal) brain growth^87^, did not demonstrate group differences in psychosis. Sulcal depth is calculated based on the Euclidian distance between the top and bottom of a sulcus, indicating that psychosis is not associated with shallower insula sulci. This is in contrast with lower gyrification, which reflects the degree of folding, but not the depth of the folds. Our data suggest that folding was reduced in psychotic disorders but the depth of the sulci were similar between groups. Previous literature on sulcal depth in psychosis is limited and mixed^43,77^. In fact, the current data may represent the most comprehensive analysis of insula sulcal depth in psychotic disorders to-date and suggests it is intact in schizophrenia and may be slightly increased in bipolar disorder. Further studies in independent cohorts are needed to replicate these findings.

Finally, sub-regional structure is associated with clinical phenotypes across the psychosis spectrum. In both cohorts, dysgranular and agranular insula volume were significantly associated with overall cognition, suggesting that smaller volume of these sub-regions contributes to greater cognitive impairment across the psychosis-spectrum – a core feature of psychosis-risk^88^. Furthermore, worse negative symptoms were associated with lower insula volume in all three sub-regions, while positive symptoms were associated with lower thickness. Insula sub-regions have distinct whole-brain connectivity patterns, reflecting their involvement in different functional systems^9,47,48,89^. Despite this, we saw limited evidence of specific brain-behavior relationships depending on insula sub-region, instead findings that volume and thickness were predictive of many of the phenotypes, particularly in the psychotic disorders cohort. This suggests that abnormal insula structure (particularly a smaller and thinner insula) contributes to overall clinical symptom severity in psychotic disorders. We do note, however, that one intriguing area of specificity was dysgranular gyrification, which was only related to cognitive ability. While requiring replication, this finding suggests that altered cortical folding driven by local connectivity contributes to the cognitive deficits observed in schizophrenia-spectrum disorders. While these effect sizes are small, in the context of large cohorts and correlational psychological research, they are still meaningful^90^ and should motivate continued research into the clinical implications of altered insula structure in psychosis.

Strengths of the current study include large sample sizes, comparison across multiple cohorts at varying stages along the psychosis-spectrum, analysis of insula sub-regions based on segmentation of granular layers, and analysis of structural integrity using both volume and surface-based measures. Limitations include different measures of cognition and symptoms collected across the cohorts, limiting direct comparison of brain-behavior relationships. Furthermore, this is a cross-sectional sample and we therefore can only infer progressive changes from psychosis-spectrum youth to psychotic disorders.

In sum, insula volume is reduced in psychotic disorders and psychosis-spectrum youth and correlates with cognitive ability in both cohorts, suggesting insula volume deficits confer broad risk for psychosis. Reduced gyrification of the insula is also observed in psychotic disorders, but is most pronounced in schizophrenia and contributes to cognitive impairment. Negative and positive symptoms are also related to insula sub-regional structure, suggesting that abnormal pathophysiology of the insula contributes to the clinical phenotypes. Future directions include longitudinal analysis of the PNC cohort to determine whether those who convert to schizophrenia demonstrate more pronounced gyrification abnormalities than the PS youth as a whole. Examination of gyrification and volume abnormalities in relationship with polygenic risk scores for schizophrenia may also reveal their contribution to cognitive and developmental subtyping of psychosis^91^. Finally, molecular analysis of granular layers in psychotic disorders can be used to assess how alterations picked up by MRI map onto the cytoarchitecture, informing biological models of psychosis.

## Supplementary information

Supplemental Material

## References

[1] Streeter, G. L. The development of the nervous system. In: *Manual of human embryology*, *vol II*. Lippincott, Philadelphia.

[2] Wylie KP, Tregellas JR (2010). The role of the insula in schizophrenia. Schizophr. Res..

[3] Goodkind M (2015). Identification of a common neurobiological substrate for mental illness. JAMA Psychiatry.

[4] Sheffield JM, Rogers BP, Blackford JU, Heckers S, Woodward ND (2020). Insula functional connectivity in schizophrenia. Schizophr. Res..

[5] Tian Y, Zalesky A, Bousman C, Everall I, Pantelis C (2019). Insula functional connectivity in schizophrenia: subregions, gradients, and symptoms. Biol. Psychiatry Cogn. Neurosci. Neuroimaging.

[6] Moran LV (2013). Disruption of anterior insula modulation of large-scale brain networks in schizophrenia. Biol. Psychiatry.

[7] Palaniyappan L, Simmonite M, White TP, Liddle EB, Liddle PF (2013). Neural primacy of the salience processing system in schizophrenia. Neuron.

[8] Nieuwenhuys, R. The insular cortex. A review. In: *Progress in Brain Research*. Elsevier B.V., 2012, pp 123–163. 10.1016/B978-0-444-53860-4.00007-6 22230626

[9] Mesulam, M. & Mufson, E. J. Insula of the Old World Monkey. I: Architectonics in the Insulo-orbito-temporal Component of the Paralimbic Brain. 10.1002/cne.902120102 7174905

[10] McDonald C (2005). Regional volume deviations of brain structure in schizophrenia and psychotic bipolar disorder: computational morphometry study. Br. J. Psychiatry.

[11] Crespo-Facorro B (2000). Insular cortex abnormalities in schizophrenia: a structural magnetic resonance imaging study of first-episode patients. Schizophr. Res.

[12] Takahashi T (2009). Diagnostic specificity of the insular cortex abnormalities in first-episode psychotic disorders. Prog. Neuro-Psychopharmacol. Biol. Psychiatry.

[13] Makris N (2006). Decreased volume of left and total anterior insular lobule in schizophrenia. Schizophr. Res..

[14] Shepherd AM, Matheson SL, Laurens KR, Carr VJ, Green MJ (2012). Systematic meta-analysis of insula volume in Schizophrenia. Biol. Psychiatry.

[15] Uddin, L. Q., Kinnison, J., Pessoa, L. & Anderson, M. L. Beyond the tripartite cognition–emotion–interoception model of the human insular cortex. 2013. 10.1162/JOCN_A_00462. 10.1162/jocn_a_00462 PMC4074004 23937691

[16] Ellison-Wright I, Bullmore E (2010). Anatomy of bipolar disorder and schizophrenia: a meta-analysis. Schizophr. Res..

[17] Rimol LM (2012). Cortical volume, surface area, and thickness in schizophrenia and bipolar disorder. Biol. Psychiatry.

[18] Lee S-H (2016). Initial and progressive gray matter abnormalities in insular gyrus and temporal pole in first-episode schizophrenia contrasted with first-episode affective psychosis. Schizophr. Bull..

[19] Bechdolf A (2012). Amygdala and insula volumes prior to illness onset in bipolar disorder: a magnetic resonance imaging study. Psychiatry Res. Neuroimaging.

[20] Takahashi T (2009). Insular cortex gray matter changes in individuals at ultra-high-risk of developing psychosis. Schizophr. Res..

[21] Smieskova R (2012). Insular volume abnormalities associated with different transition probabilities to psychosis. Psychol. Med..

[22] Borgwardt SJ (2007). Regional gray matter volume abnormalities in the at risk mental state. Biol. Psychiatry.

[23] Ellison-Wright I, Glahn DC, Laird AR, Thelen SM, Bullmore E (2008). The anatomy of first-episode and chronic schizophrenia: an anatomical likelihood estimation meta-analysis. Am. J. Psychiatry.

[24] Chan RCK, Di X, McAlonan GM, Gong Q-Y (2011). Brain anatomical abnormalities in high-risk individuals, first-episode, and chronic schizophrenia: an activation likelihood estimation meta-analysis of illness progression. Schizophr. Bull..

[25] Mills KL (2016). Structural brain development between childhood and adulthood: convergence across four longitudinal samples. Neuroimage.

[26] Giedd JN, Rapoport JL (2010). Structural MRI of pediatric brain development: what have we learned and where are we going?. Neuron.

[27] Shaw, P. et al. Neurodevelopmental Trajectories of the Human Cerebral Cortex. 2008. 10.1523/JNEUROSCI.5309-07.2008. 10.1523/JNEUROSCI.5309-07.2008 PMC6671079 18385317

[28] Shaw P (2006). Intellectual ability and cortical development in children and adolescents. Nature.

[29] Giedd JN (2009). Anatomical brain magnetic resonance imaging of typically developing children and adolescents. J. Am. Acad. Child Adolesc. Psychiatry.

[30] Rakic P (1988). Specification of cerebral cortical areas. Science.

[31] Norbom, L. B. et al. New insights into the dynamic development of the cerebral cortex in childhood and adolescence: integrating macro- and microstructural MRI findings. 10.31234/OSF.IO/F384C. 10.1016/j.pneurobio.2021.102109 34147583

[32] Hofer E (2020). Genetic correlations and genome-wide associations of cortical structure in general population samples of 22,824 adults. Nat. Commun..

[33] Armstrong E, Schleicher A, Omran H, Curtis M, Zilles K (1995). The ontogeny of human gyrification. Cereb. Cortex.

[34] Zilles K, Palomero-Gallagher N, Amunts K (2013). Development of cortical folding during evolution and ontogeny. Trends Neurosci..

[35] Meng Y, Li G, Lin W, Gilmore JH, Shen D (2014). Spatial distribution and longitudinal development of deep cortical sulcal landmarks in infants. Neuroimage.

[36] Im K, Grant PE (2019). Sulcal pits and patterns in developing human brains. Neuroimage.

[37] Ducharme S (2016). Trajectories of cortical thickness maturation in normal brain development - The importance of quality control procedures. Neuroimage.

[38] Remer J (2017). Quantifying cortical development in typically developing toddlers and young children, 1–6 years of age. Neuroimage.

[39] Roiz-Santiáñez R (2010). Insular cortex thinning in first episode schizophrenia patients. Psychiatry Res. Neuroimaging.

[40] Spalthoff R, Gaser C, Nenadić I (2018). Altered gyrification in schizophrenia and its relation to other morphometric markers. Schizophr. Res..

[41] Nesvåg R (2008). Regional thinning of the cerebral cortex in schizophrenia: effects of diagnosis, age and antipsychotic medication. Schizophr. Res..

[42] Palaniyappan L, Liddle PF (2012). Aberrant cortical gyrification in schizophrenia: a surface-based morphometry study. J. Psychiatry Neurosci..

[43] Yan J (2019). Cortical thinning and flattening in schizophrenia and their unaffected parents. Neuropsychiatr. Dis. Treat..

[44] Palaniyappan L, Mallikarjun P, Joseph V, White TP, Liddle PF (2011). Reality distortion is related to the structure of the salience network in schizophrenia. Psychol. Med..

[45] Cauda F (2012). Meta-analytic clustering of the insular cortex: characterizing the meta-analytic connectivity of the insula when involved in active tasks. Neuroimage.

[46] Howes OD, Murray RM (2014). Schizophrenia: an integrated sociodevelopmental-cognitive model. Lancet.

[47] Evrard HC (2019). The organization of the primate insular cortex. Front. Neuroanat..

[48] Deen B, Pitskel NB, Pelphrey KA (2011). Three systems of insular functional connectivity identified with cluster analysis. Cereb. Cortex.

[49] Chang LJ, Yarkoni T, Khaw MW, Sanfey AG (2013). Decoding the role of the insula in human cognition: functional parcellation and large-scale reverse inference. Cereb. Cortex.

[50] Kelly C (2012). A convergent functional architecture of the insula emerges across imaging modalities. Neuroimage.

[51] Uddin LQ, Nomi JS, Hébert-Seropian B, Ghaziri J, Boucher O (2017). Structure and function of the human insula. J. Clin. Neurophysiol..

[52] Calkins ME (2015). The Philadelphia Neurodevelopmental Cohort: constructing a deep phenotyping collaborative. J. Child Psychol. Psychiatry.

[53] Kay, S. R., Opler, L. A. & Lindenmayer, J. P. The Positive and Negative Syndrome Scale (PANSS): rationale and standardisation. *Br. J. Psychiatry* **155**, 59–67 (1989). 2619982

[54] Purdon, S. E. *The Screen for Cognitive Impairment in Psychiatry (SCIP): Administration Manual and Normative Data*. PNL Inc: Edmonton, Alberta, 2005.

[55] Gomez-Benito J (2013). The screen for cognitive impairment in psychiatry: diagnostic-specific standardization in psychiatric ill patients. BMC Psychiatry.

[56] Kay SR, Fiszbein A, Opler LA (1987). The positive and negative syndrome scale (PANSS) for schizophrenia. Schizophr. Bull..

[57] Satterthwaite TD (2015). Connectome-wide network analysis of youth with Psychosis-Spectrum symptoms. Mol. Psychiatry.

[58] Wolf DH (2015). Functional neuroimaging abnormalities in youth with psychosis spectrum symptoms. JAMA Psychiatry.

[59] Gur RC (2012). Age group and sex differences in performance on a computerized neurocognitive battery in children age 8–21. Neuropsychology.

[60] Friston, K. J. et al. Statistical parametric maps in functional imaging: a general linear approach. *Hum Brain Mapp* 1994. 10.1002/hbm.460020402.

[61] Dahnke, R., Ziegler, G., & Gaser C. Local adaptive segmentation. Beijing, HBM. 2012.

[62] Dahnke R, Yotter RA, Gaser C (2013). Cortical thickness and central surface estimation. Neuroimage.

[63] Luders E (2006). A curvature-based approach to estimate local gyrification on the cortical surface. Neuroimage.

[64] Farb NAS, Segal ZV, Anderson AK (2013). Attentional modulation of primary interoceptive and exteroceptive cortices. Cereb. Cortex.

[65] Brett M., Anton J.-L., Valabregue, R. & Poline, J.-B. Region of interest analysis using an SPM toolbox. Sendai, Japan, (2002) http://www.mrc-cbu.cam.ac.uk/Imaging/marsbar.html (Accessed 15 May 2020).

[66] Glasser MF (2016). A multi-modal parcellation of human cerebral cortex. Nature.

[67] Desikan RS (2006). An automated labeling system for subdividing the human cerebral cortex on MRI scans into gyral based regions of interest. Neuroimage.

[68] Augustine, J. R. Circuitry and functional aspects of the insular lobe in primates including humans. *Brain Res. Rev*. 10.1016/S0165-0173(96)00011-2 (1996). 10.1016/s0165-0173(96)00011-2 8957561

[69] Craig, A. D. How do you feel—now? The anterior insula and human awareness. Nat. Rev. Neurosci. 10, 59–70 (2009). 10.1038/nrn2555 19096369

[70] Uddin LQ (2015). Salience processing and insular cortical function and dysfunction. Nat. Rev. Neurosci..

[71] Wang X (2019). Brain grey-matter volume alteration in adult patients with bipolar disorder under different conditions: a voxel-based meta-analysis. J. Psychiatry Neurosci..

[72] Fusar-Poli P (2012). Predicting psychosis: meta-analysis of transition outcomes in individuals at high clinical risk. Arch. Gen. Psychiatry.

[73] Palaniyappan L, Liddle PF (2014). Diagnostic discontinuity in psychosis: a combined study of cortical gyrification and functional connectivity. Schizophr. Bull..

[74] Nesvåg R (2014). Reduced brain cortical folding in schizophrenia revealed in two independent samples. Schizophr. Res..

[75] Afif A, Bouvier R, Buenerd A, Trouillas J, Mertens P (2007). Development of the human fetal insular cortex: study of the gyration from 13 to 28 gestational weeks. Brain Struct. Funct..

[76] White T, Su S, Schmidt M, Kao CY, Sapiro G (2010). The development of gyrification in childhood and adolescence. Brain Cogn..

[77] Damme KSF (2019). Cortical morphometry in the psychosis risk period: a comprehensive perspective of surface features. Biol. Psychiatry Cogn. Neurosci. Neuroimaging.

[78] Van Essen DC (1997). A tension-based theory of morphogenesis and compact wiring in the central nervous system. Nature.

[79] White, T. & Hilgetag, C. C. Gyrification and neural connectivity in schizophrenia 10.1017/S0954579410000842. (2006). 10.1017/S0954579410000842 21262059

[80] Khadka S (2013). Is aberrant functional connectivity a psychosis endophenotype? A resting state functional magnetic resonance imaging study. Biol. Psychiatry.

[81] Sheffield, J. M., Karcher, N. R. & Barch, D. M. Cognitive deficits in psychotic disorders: a lifespan perspective. *Neuropsychol. Rev.* **28**10.1007/s11065-018-9388-2 (2018). 10.1007/s11065-018-9388-2 PMC6475621 30343458

[82] Huttenlocher PR (1990). Morphometric study of human cerebral cortex development. Neuropsychologia.

[83] Sowell ER (2004). Longitudinal mapping of cortical thickness and brain growth in normal children. J. Neurosci..

[84] Li G, Lin W, Gilmore JH, Shen D (2015). Spatial patterns, longitudinal development, and hemispheric asymmetries of cortical thickness in infants from birth to 2 years of age. J. Neurosci..

[85] Winkler AM (2018). Joint analysis of cortical area and thickness as a replacement for the analysis of the volume of the cerebral cortex. Cereb. Cortex.

[86] Palaniyappan L, Liddle PF (2012). Differential effects of surface area, gyrification and cortical thickness on voxel based morphometric deficits in schizophrenia. Neuroimage.

[87] Im, K. et al. Reliable identification of deep sulcal pits: the effects of scan session, scanner, and surface extraction tool. *PLoS ONE* **8**10.1371/journal.pone.0053678 (2013). 10.1371/journal.pone.0053678 PMC3538732 23308272

[88] Reichenberg A (2005). Cognitive impairment as a risk factor for psychosis. Dialogues Clin. Neurosci..

[89] Namkung H, Kim S-H, Sawa A (2017). The insula: an underestimated brain area in clinical neuroscience, psychiatry, and neurology. Trends Neurosci..

[90] Funder DC, Ozer DJ (2019). Evaluating effect size in psychological research: sense and nonsense. Adv. Methods Pr. Psychol. Sci..

[91] Dickinson D (2020). Distinct polygenic score profiles in schizophrenia subgroups with different trajectories of cognitive development. Am. J. Psychiatry.

