# Supplementary material for: Insula sub-regions across the psychosis spectrum: morphology and clinical correlates
Source: Transl Psychiatry. 2021 Jun 4;11:346. doi: 10.1038/s41398-021-01461-0 (PMC8178380; doi:10.1038/s41398-021-01461-0)
Supplement: Supplementary file 1 — Supplemental Material [file 41398_2021_1461_MOESM1_ESM.pdf]

Supplemental Information

Methods

Study Procedures: Psychosis Cohort

Individuals with a psychotic disorder were recruited from the psychiatric inpatient and outpatient clinics of the Vanderbilt University Medical Center Psychotic Disorders Program and healthy individuals were recruited from Nashville and the surrounding community via advertisements and word of mouth. Study procedures were approved by the Vanderbilt Institutional Review Board. Informed consent was obtained from all study participants.

Diagnoses were confirmed using the Structured Clinical Interview for DSM-IV (SCID) (1). Premorbid IQ was estimated with the Wechsler Test of Adult Reading (WTAR) (2). Exclusion criteria were similar across all three studies. Participants were excluded for: significant medical or neurological illness, head injury, pregnancy, age <16 and >65, premorbid IQ less than 70, substance abuse or dependence (within the last one month in patients, lifetime in healthy individuals), and any MRI contra-indicators (e.g. metal implants, claustrophobia). Healthy individuals were excluded if they had current or past psychiatric illness or a first degree relative with a psychotic illness. Inclusion criteria differed slightly across studies, namely a lower age range in R01MH102266 (18 to 55), and a slightly longer duration of substance abuse/dependence remission (no substance use disorder in past three months in psychosis, no lifetime history of substance use disorder in healthy controls).

Of the 602 participants, 103 were excluded from the study for at least one of the following reasons (25 met multiple exclusion criteria): 17 were found ineligible based on inclusion criteria, 73 were excluded based on visual inspection of CAT12 segmentation data (blinded by group), 40 were excluded based on visual inspection of structural data for poor gray matter segmentation, 28 were determined to have unusable T1 scans based on excessive motion and/or artefacts, and 5 were excluded for identifying as significant outliers in CAT12 for VBM (N=2) or topology defects (N=3).

Cognitive ability was measured using the Screen of Cognitive Impairment in Psychiatry (SCIP), consisting of five sub-tests: verbal memory (immediate and delayed), working memory, verbal

fluency, and processing speed. SCIP subtest SCIP subtest raw scores were converted to z-scores using normative data and averaged to create a composite z-score (3). Symptom severity was assessed using the Positive and Negative Syndrome Scale, which rates positive, negative and general psychopathology symptoms over the past two weeks (4). Average positive and negative scale scores were used to measure positive and negative symptom severity.

Study Procedures: Philadelphia Neurodevelopmental Cohort

The Philadelphia Neurodevelopmental Cohort (PNC) is a community cohort, as opposed to a high-risk, help seeking sample, providing a wider range of symptom severity and overall less severe symptoms. Study procedures were approved by the Institutional Review Boards of the University of Pennsylvania and the Children's Hospital of Pennsylvania (CHOP) and informed consent was obtained from all study participants. All participants completed a clinical assessment that included structured interviews and rating scales as described previously. In brief, study participants were assessed with the GOASSES, a computerized structured clinical interview that includes collecting demographic data, a timeline of life events, medical history, and an abbreviated version of the Schedule for Affective Disorders and Schizophrenia for School-Age Children (K-SADS) to assess a range of psychopathology. In addition, psychotic symptoms were more thoroughly assessed using the PRIME Screen-Revised (PS-R), K-SADS psychosis screen, and selected items from the Scale for Prodromal Syndromes (SOPS). The PS-R consists of 12 self-rated items assessing sub-threshold positive symptoms experienced in the past year. Each item is rated on a 7-point scale where 0=Definitely Disagree and 6=Definitely Agree and a total score was calculated by summing the 12 items. The K-SADS psychosis screen assesses for positive psychosis symptoms—hallucinations and delusions specifically. Negative/disorganized items administered from the SOPS included: N2 Avolition, N3 Expression of Emotion, N4 Experience of Emotions and Self, N6 Occupational Functioning, D3 Trouble with Focus and Attention, and P5 Disorganized Communication. A SOPS total score was calculated by summing the 6 items.

Youth were classified as psychosis spectrum using the same criteria as previously described (5). Briefly, individuals were classified as psychosis spectrum if they; 1) had an age-deviant PRIME total score >=2 SD above age-matched peers or had one or more PRIME items rated 6, or 3 or more items rated 5; 2) endorsed definite or possible hallucinations or delusions on the K-

SADS psychosis screen; or 3) had an age-deviant SOPS negative/disorganized score >=2 SD above age-matched peers.

Youth with other psychopathologies were defined as those that had sub-threshold psychopathology symptoms—endorsed symptoms, frequency, and duration approximate with DSM-IV disorders or episode criteria, with significant distress (≥5) on the K-SADS—but did not meet psychosis criteria.

Typically developing youth were defined as those that did not meet criteria for either psychosis or other psychopathologies.

Of the 1601 participants with imaging data, 69 were excluded for serious medical conditions, 19 for a diagnosis of autism, 63 for insufficient clinical data to reach a diagnosis and 84 for poor scan and/or segmentation quality. A small number of individuals from each group were taking psychotropic medications (typically developing: n=16; psychosis spectrum: n=71; other psychopathologies: n=49).

In the PNC, cognitive ability was measured using the Penn Computerized Neurocognitive Battery (6), which consists of 14 tests covering five cognitive domains: executive function, episodic memory, social cognition, complex cognition, and sensorimotor ability. A general cognitive ability score was created by averaging accuracy z-scores across the four non-motor domains.

PNC Sensitivity Analysis

Differences in age, sex and race were notable between the three groups. In order to determine the impact of these variables on our significant results, we ran a sensitivity analysis by creating sub-samples of the psychosis spectrum and typically developing youth matched for age, sex and race. Matching was conducted using the MatchIt package (Version 3.0.2) in R (Version 3.6.1; R Core Team, 2019), using the 'exact' matching method, which matches each member of the typically developing youth with any psychosis spectrum youth that have the same values for age, sex and race. Race was considered as a binary variable, separated into Caucasian and non-Caucasian youth. This resulted in a final sample of 143 psychosis spectrum and 120 typically developing youth. See Table S10 for demographics.

MRI Acquisition

Psychosis cohort: Neuroimaging data were acquired on two identical 3T Philips Intera Achieva scanners (32 channel received head coil, single-band imaging) located at the Vanderbilt Institute for Imaging Sciences (VUIIS). In all three studies, a high resolution T1-weighted structural scans were collected with a 3D T1 fast field echo sequence with 1 mm^3 isotropic voxels (TR/TE = 8.0/3.7, FOV=256 x 256 x 170 mm, matrix = 256 x 256 x 170, flip angle = 5°).

PNC: High resolution T1-weighted structural scans were acquired with the MPRAGE sequence on a Siemens Tim Trio 3T scanner with a 32-channel head coil with 0.93 x 0.93 x 1 mm voxels (160 slices, TR/TE = 1810/3.5, FOV = 180 x 240 x 160, matrix = 192 x 256 x 160; flip angle = 9°).

Voxel-Based Morphometry Quality Assurance

Segmented gray matter images were modulated by the normalization factors to preserve the original gray matter volume. CAT12 provides a quantitative measure of image quality that evaluates image parameters such as noise, inhomogeneities and image resolution. This measure is placed on a rating scale and assigned a letter grade, with any scan rated B- or above considered good quality and scans rated C- to C+ considered satisfactory. In our data, scans rated C+ or below were further visually inspected, blind to group membership, for gray matter segmentation quality. Scans with gray matter segmentation that did not capture the gray matter, or included non-gray matter voxels (e.g. skull), were excluded from further analysis. Additionally, the modulated, normalized gray matter segmentations were checked using the CAT12 automated quality check protocol, which checks image inhomogeneity, defined as the mean correlation between gray matter volumes, with higher correlation indicating greater homogeneity. Flagged images were visually inspected, and segmented images with significant inhomogeneity were excluded from further analysis. The CAT12 pipeline was containerized using Singularity, built at SingularityHub (7) https://singularity-hub.org), and executed using the Vanderbilt University Institute of Imaging Science XNAT infrastructure (8).

Surface-Based Morphometry

Project-based thickness (PBT) uses a tissue segmentation to estimate white matter distance and then projects the local maxima onto gray matter voxels using a neighboring relationship (10). Topological defects of the surface reconstruction are corrected using a method based on spherical harmonics, in which the original MRI intensity values are used to identify and “fill” or “cut” each topological defect. These defects are patched using a low-pass filtered alternative reconstruction (12). The number of topological defects identified within-subject is used as a covariate in all surface-based analyses, following the removal of subjects with $z>3.0$ defects. To enable inter-subject analysis, a spherical map of a cortical surface is used to reparameterize the surface mesh into a common coordinate system (13). Finally, spherical registration is conducted by applying a multi-grid approach that uses reparameterized values of sulcal depth and shape index defined on the sphere (14).

Regarding the SBM measures calculated in CAT12, cortical thickness estimates the thickness of the gray matter surface. Gyrification estimates the cortical folding within a region of interest based on absolute mean curvature, with greater cortical folding contributing to greater local gyrification. Square-root transformed values of sulcal depth were also estimated in CAT12 based on the Euclidean distance between the central surface and its convex hull.

Insula sub-region definition

Insula volume was calculated for each sub-region based on masks provided by Farb and colleagues (9). These masks include hand-drawn insula parcellations based on well-characterized cytoarchitectonic divisions, which comprised the agranular, dysgranular and granular sub-regions. The mask was defined on a high-resolution T1-weighted template, based on well-characterized cytoarchitectonic divisions (15). Insula surface metrics were calculated based on the HCP-MMP1 atlas based on the guidance by Glasser (11). The HCP-MMP1 sub-region designations were as follows: Agranular = AVI & AAIC; Dysgranular = PoI1, PoI2, & MI; Granular = Ig.

Results

Insula Sub-Region Differences

Prior to analyzing group differences, we characterized differences in morphology (thickness, gyrification, sulcal depth) across insula sub-regions. Volume was excluded from this analysis due to differences in the sizes of the a-priori insula ROIs. A significant main effect of region was observed for all SBM metrics, in both the psychosis and PNC cohorts (all $p<.001$). The pattern of regional differences for each metric was the same across cohorts. Thickness: dysgranular>agranular>granular; Gyrification: granular>agranular>dysgranular; Sulcal Depth: granular>dysgranular>agranular. Interestingly, all of the patterns differed from one another, highlighting their relative independence in indexing different aspects of insula structure (full data in Table S1).

Illness Stage Effects

To determine the impact of illness stage on insula volume, patients were divided into early psychosis (illness duration ≤ 2 years; N=146) and chronic psychosis (illness duration >2 years; N=147).

For volume, there was a main effect of group ($F(2,484)=9.59$, $p<.001$) and region ($F(2,968)=13.42$, $p<.001$) but a non-significant group by region interaction ($F(4,968)=1.94$, $p=.145$). The main effect of group was driven by significantly lower insula volume in all three sub-regions in early (agranular: 5.8% smaller, $p=.001$; dysgranular: 4.4% smaller, $p=.008$; granular: 3.9% smaller, $p=.006$) and chronic (agranular: 5.9% smaller, $p<.001$; dysgranular: 5.2% smaller, $p=.001$; granular: 4.4% smaller, $p=.001$) patients compared to controls.

Cortical thickness was similar between groups ($F(2,483)=.15$, $p=.857$) and there was a non-significant group by region interaction ($F(4,966)=2.17$, $p=.07$). Gyrification was significantly different between groups ($F(2,483)=4.08$, $p=.017$) but there was a non-significant group by region interaction ($F(4,966)=1.41$, $p=.229$). Both early and chronic patients had reduced gyrification compared to healthy controls, however this difference was significant only for early psychosis ($p=.007$), not chronic ($p=.103$) and was significant only at the whole-insula level (i.e. not within sub-region). Sulcal depth did not differ between groups ($F(2,483)=1.16$, $p=.316$). The group by region interaction did not reach statistical significance ($F(4,966)=2.31$, $p=.056$).

Diagnostic Specificity (Schizophrenia-Spectrum vs. Psychotic Bipolar Disorder)

Volume: There was a main effect of group ($F(2,492)=11.90$, $p<.001$) and a main effect of region ($F(2,984)=13.522$, $p<.001$), but no significant group by region interaction ($F(4,984)=1.83$, $p=.121$). Overall insula volume was significantly smaller in schizophrenia participants compared to healthy (5.75% smaller, $p<.001$) and bipolar participants (3.97% smaller, $p=.01$). Bipolar participants did not significantly differ from healthy participants (1.86% smaller, $p=.241$). This pattern held across all three sub-areas, with schizophrenia participants demonstrating significant reductions in insula volume compared to both groups. Healthy and bipolar participants were not significantly different from one another.

Thickness: For cortical thickness, there was a main effect of region ($F(2,982)=22.92$, $p<.001$) but no significant main effect of group ($F(2,491)=2.06$, $p=.128$) or group by region interaction ($F(4,982)=1.25$, $p=.29$).

Gyrification: When considering schizophrenia, bipolar and healthy participants, there was a significant main effect of group ($F(2,491)=3.90$, $p=.021$), region ($F(2,982)=25.69$, $p<.001$), and a significant group by region ($F(4,982)=2.79$, $p=.025$) interaction for gyrification. Whole insula gyrification was significantly reduced in schizophrenia compared to healthy participants (1.19% reduced, $p=.005$). Bipolar participants did not differ from either group (p's$>.25$). Overall groups differences for sub-regions were: agranular ($p=.104$), dysgranular ($p=.009$) and granular ($p=.046$). After correcting for multiple comparisons, the only significant pairwise difference was reduced gyrification of the dysgranular insula in schizophrenia compared to healthy controls.

Sulcal Depth: There was a significant main effect of region ($F(2,982)=45.83$, $p<.001$), group ($F(2,491)=5.00$, $p=.007$), and a group by region interaction ($F(4,982)=3.032$, $p=.017$). This difference was driven by larger sulcal depth in bipolar participants compared to schizophrenia (1.01% greater, $p=.002$) and healthy participants (.71% greater, $p=.028$). All sub-regions differed between groups (agranular: $p=.01$; dysgranular: $p=.026$; granular: $p=.003$). Pairwise comparisons showed significantly greater sulcal depth for bipolar participants compared to schizophrenia for all three regions, and greater sulcal depth compared to controls for the agranular insula.

Sensitivity Analysis for PNC volume results

To further mitigate the potential impact of demographic variables (age, gender, race) on the volume findings, we conducted a sensitivity analysis on a smaller group of PS (N=143) and TD (N=126) youth matched on demographic features using the MatchIt package in R (16) (Table S10). While the group differences were no longer statistically significant in the much smaller samples (F(1,226)=1.54, p=.216), the magnitude of difference between the groups was nearly identical to what was observed in the larger sample for agranular (1.6% smaller, p=.097) and dysgranular (1.2% smaller, p=.178) regions. The granular insula demonstrated similar volume between groups (p=.962).

Age Effect Analyses

Age effects were examined in separate linear regression models with insula sub-area structural metrics as the outcome variables, age and age by group interactions as the predictors. Sex, TIV, topography defects (surface-metrics) and project (psychosis cohort) were included as covariates. Age effects are visualized in Figures S2 and S3.

Psychosis Cohort: Volume and sulcal depth were strongly negatively associated with age for all insula sub-areas. Results were more variable for cortical thickness and gyrification (full results presented in Table S7). No significant group by age interactions were observed for any metrics except dysgranular insula gyrification (p=.002). Investigation into this interaction revealed a significant positive association between age and dysgranular gyrification in healthy controls (r=.19, p=.009) that was not observed in psychosis patients (r=-.06, p=.303).

PNC Cohort: Volume and sulcal depth were negatively associated with age for all insula sub-regions, similar to the psychosis cohort, with more variable associations for thickness and gyrification (Table S8). The only significant group by age interaction across all metrics was between OP and TD youth for granular gyrification. In the TD youth, age and granular gyrification were negatively associated (r=-.10, p=.042) while they were positively associated in OP youth (r=.11, p=.005).

Sex Effects Analysis

Main effects and group by sex interactions were largely non-significant within both cohorts (Tables S7-S8). In the psychosis cohort, women had slightly greater granular gyrification than men ($p=.003$). In the PNC, boys had slightly greater dysgranular volume ($p=.007$), dysgranular thickness ($p<.001$), and agranular gyrification ($p=.001$) than girls.

References

1. First MB, Spitzer RL, Gibbon M, Williams JBW (1995): *Structured Clinical Intervew for DSM-IV Axis I Disorders- Non-Patient Edition (SCID-I/NP, Version 2.0)*. (B. R. Department, editor). New York.
2. Weschler D (2001): *Weschler Test of Adult Reading (WTAR)*. (T. P. Corporation, editor). San Antonio, TX.
3. Purdon SE (2005): *The Screen for Cognitive Impairment in Psychiatry (SCIP): Administration Manual and Normative Data*. Edmonton, Alberta: PNL Inc.
4. Kay SR, Fiszbein A, Opler LA (1987): The positive and negative syndrome scale (PANSS) for schizophrenia. *Schizophr Bull*, 1987/01/01. 13: 261–276.
5. Satterthwaite TD, Vandekar SN, Wolf DH, Bassett DS, Ruparel K, Shehzad Z, *et al.* (2015): Connectome-wide network analysis of youth with Psychosis-Spectrum symptoms. *Mol Psychiatry*, 2015/06/03. 20: 1508–1515.
6. Gur RC, Richard J, Calkins ME, Chiavacci R, Hansen JA, Bilker WB, *et al.* (2012): Age group and sex differences in performance on a computerized neurocognitive battery in children age 8-21. *Neuropsychology*. 26: 251–265.
7. Sochat V V., Prybol CJ, Kurtzer GM (2017): Enhancing reproducibility in scientific computing: Metrics and registry for Singularity containers. *PLoS One*. . doi: 10.1371/journal.pone.0188511.
8. Harrigan RL, Yvernault BC, Boyd BD, Damon SM, Gibney KD, Conrad BN, *et al.* (2016): Vanderbilt University Institute of Imaging Science Center for Computational Imaging XNAT: A multimodal data archive and processing environment. *Neuroimage*. . doi: 10.1016/j.neuroimage.2015.05.021.
9. Farb NAS, Segal Z V., Anderson AK (2013): Attentional Modulation of Primary Interoceptive and Exteroceptive Cortices. *Cereb Cortex*. 23: 114–126.
10. Gaser C, Dahnke R (2016): CAT-a computational anatomy toolbox for the analysis of structural MRI data. *HBM*. 336–348.
11. Glasser MF, Coalson TS, Robinson EC, Hacker CD, Harwell J, Yacoub E, *et al.* (2016): A multi-modal parcellation of human cerebral cortex. *Nature*. 536: 171–178.
12. Yotter RA, Dahnke R, Thompson PM, Gaser C (2011): Topological correction of brain surface meshes using spherical harmonics. *Hum Brain Mapp*. 32: 1109–1124.
13. Yotter RA, Thompson PM, Gaser C (2011): Algorithms to Improve the Reparameterization of Spherical Mappings of Brain Surface Meshes. *J Neuroimaging*. 21: e134–e147.
14. Ashburner J (2007): A fast diffeomorphic image registration algorithm. *Neuroimage*. 38: 95–

113.

15. Mesulam M, Mufson EJ (1982): *Insula of the Old World Monkey. I: Architectonics in the Insulo-orbito-temporal Component of the Paralimbic Brain*. *J Comp Neurol*. (Vol. 2). Retrieved May 22, 2019, from https://onlinelibrary.wiley.com/doi/pdf/10.1002/cne.902120102.

16. (No Title) (n.d.): . Retrieved March 6, 2020, from https://r.iq.harvard.edu/docs/matchit/2.4-18/matchit.pdf.

Table S1: Main effect of sub-region within each cohort: marginal means (standard error)

Psychosis Cohort					PNC Cohort			
	**Agranular**	**Dysgranular**	**Granular**	**Main Effect**	**Agranular**	**Dysgranular**	**Granular**	**Main Effect**
**Cortical Thickness**	3.52 (.012)	3.57 (.013)	3.01 (.011)	F(2,984)=23.06, p<.001	3.65 (.009)	3.73 (.01)	3.19 (.008)	F(2,2722)=9.32, p<.001
**Gyrification**	26.50 (.067)	25.05 (.071)	29.581 (.083)	F(2,984)=27.29, p<.001	27.03 (.04)	24.86 (.044)	29.77 (.05)	F(2,2722)=77.09, p<.001
**Sulcal Depth**	4.70 (.005)	4.79 (.007)	5.25 (.006)	F(2,984)=45.89, p<.001	4.74 (.003)	4.83 (.004)	5.30 (.004)	F(2,2722)=161.97, p<.001

Table S2: Full statistics for vertex- and voxel-based analyses

	**Montreal Neurological Coordinates (x,y,z)**	**Peak t-value**	$p_{FWE\text{-}corrected}$	**Cluster Size (Voxels)**		**Montreal Neurological Coordinates (x,y,z)**	**Peak F-value**	$p_{FWE\text{-}corrected}$	**Cluster Size (Voxels)**
**Psychosis Cohort (Healthy Controls > Psychosis)**					**PNC Cohort (3 groups)**				
Volume					*Volume*				
	-33, 8, -12	5.52	<.001	1166		38,15, -10	8.62	.063	33
	44, -10, -2	5.32	<.001	800					
Cortical Thickness					*Cortical Thickness*				
	-54, 7, 0	4.49	0.02	525		--	--	--	--
Gyrification					*Gyrification*				
	36, -21, 3	4.43	.006	806		--	--	--	--
	-35, -9, 11	3.42	.054	232					
Sulcal Depth					*Sulcal Depth*				
	--	--	--	--		--	--	--	--

Table S3: Group differences in insula structure in schizophrenia-spectrum and psychotic bipolar disorder

**Volume**	**Agranular**	**Dysgranular**	**Granular**
**HC>SZ**	6.44%, p<.001*	5.6%, p<.001*	4.74%, p<.001*
**BP>SZ**	3.76%, p=.04	3.98%, p=.018	3.98%, p=.005*
**HC > BP**	2.79%, p=.114	1.67%, p=.33	0.79%, p=.596
**Gyrification**			
**HC>SZ**	.60%, p=.281	1.87%, p=.002*	1.14%, p=.061
**BP>SZ**	1.47%, p=.035	1.16%, p=.14	-.63%, p=.420
**HC > BP**	-.009%, p=.216	.72%, p=.366	.72%, p=.366
**Sulcal Depth**			
**HC>SZ**	.11%, p=.613	.12%, p=.654	.61%, p=.023
**BP>SZ**	.84%, p=.003*	1.06%, p=.008*	1.1%, p=.001*
**HC>BP**	-.74%, p=.012*	-.93%, p=.024	-.49%, p=.146

Table S4: Brain-behavior correlations between insula sub-region morphology and clinical phenotypes in the Psychosis and PNC cohorts

Psychosis Cohort												
	Agranular				Dysgranular				Granular			
	Volume	Thickness	Gyrif	SD	Volume	Thickness	Gyrif	SD	Volume	Thickness	Gyrif	SD
Cognition	r=.22* p<.001	r=.19* p<.001	r=.08 p=.087	r=.08 p=.098	r=.20* p<.001	r=.18* p<.001	r=.12* p=.01	r=.01 p=.766	r=.21* p<.001	r=.15* p<.001	r=.07 p=.13	r=.07 p=.104
Negative	r=-.19* p<.001	r=-.19* p=.001	r=-.07 p=.269	r=.00 p=.958	r=-.18* p=.002	r=-.16 p=.008	r=- .023 p=.701	r=.03 p=.562	r=-.18* p=.002	r=-.10 p=.08	r=-.04 p=.528	r=.02 p=.769
Positive	r=-.16 p=.008	r=-.17* p=.006	r=.01 p=.916	r=-.02 p=.759	r=-.14 p=.02	r=-.14 p=.014	r=-.05 p=.369	r=.05 p=.364	r=-.11 p=.07	r=-.15* p=.01	r=.01 p=.842	r=.04 p=.523
**PNC Cohort**												
	Agranular				Dysgranular				Granular			
	Volume	Thickness	Gyrif	SD	Volume	Thickness	Gyrif	SD	Volume	Thickness	Gyrif	SD
Cognition	r=.11* p<.001	r=.01 p=.685	r=-.03 p=.322	r=.15* p<.001	r=.12* p<.001	r=-.004 p=.886	r=.06 p=.04	r=.12* p<.001	r=.04 p=.14	r=.002 p=.949	r=.06 p=.03	r=.09* p<.001
Negative	r=-.003 p=.93	r=-.02 p=.386	r=.03 p=.248	r=-.01 p=.786	r=-.017 p=.536	r=-.03 p=.361	r=-.05 p=.07	r=.02 p=.538	r=-.04 p=.15	r=.01 p=.615	r=-.01 p=.676	r=.03 p=.281
Positive	r=-.04 p=.10	r=-.001 p=.97	r=.08* p=.004	r=-.03 p=.285	r=.00 p=.996	r=.04 p=.125	r=-.03 p=.338	r=-.02 p=.462	r=.01 p=.692	r=.01 p=.825	r=.02 p=.505	r=-.02 p=.46

Two-tailed partial correlations controlling for age, gender, total intracranial volume (TIV), group, and topography (surface-based measures). *significant p-value after Bonferroni correction. Gray shading indicates correlations that were *a-priori* hypothesized.

Table S5: Age Effects: Psychosis Cohort

Volume	Statistics	
	$R_{partial}{}^{a}$	p
**Main Effect of Age**		
Agranular	-.300	<.001
Dysgranular	-.333	<.001
Granular	-.370	<.001
**Age by Group Interaction**		
Agranular	.057	.208
Dysgranular	.062	.167
Granular	.052	.250
**Cortical Thickness**	**Statistics**	
	$R_{partial}{}^{a}$	p
**Main Effect of Age**		
Agranular	-.103	.022
Dysgranular	-.074	.100
Granular	-.137	.002
**Age by Group Interaction**		
Agranular	.025	.575
Dysgranular	.052	.251
Granular	.077	.089
**Gyrification**	**Statistics**	
	$R_{partial}{}^{a}$	p
**Main Effect of Age**		
Agranular	.167	<.001
Dysgranular	.034	.450
Granular	-.054	.232
**Age by Group Interaction**		
Agranular	-.016	.717
Dysgranular	.140	.002

Granular	.036	.419
**Sulcal Depth**	**Statistics**	
	**$R_{partial}$[a]**	**p**
**Main Effect of Age**		
Agranular	-.321	<.001
Dysgranular	-341	<.001
Granular	-.285	<.001
**Age by Group Interaction**		
Agranular	.023	.605
Dysgranular	-.026	.572
Granular	.008	.855

Table S6: Age effects: PNC Cohort

**Volume**	**Statistics[a]**			
	$\mathbf{R_{partial}}$	**p**	$\mathbf{R_{partial}}$	**p**
**Main Effect of Age**				
Agranular	-.377	<.001	--	--
Dysgranular	-.480	<.001	--	--
Granular	-.510	<.001	--	--
**Age by Group Interactions**				
	Typically Developing by Psychosis Spectrum		Typically Developing by Other Psychopathologies	
Agranular	-.026	.343	-.026	.346
Dysgranular	.041	132	.027	.327
Granular	.048	.075	.011	.672
**Cortical Thickness**	**Statistics[a]**			
	$\mathbf{R_{partial}}$	**p**	$\mathbf{R_{partial}}$	**p**
**Main Effect of Age**				
Agranular	.116	<.001	--	--
Dysgranular	.123	<.001	--	--
Granular	-.142	<.001	--	--
**Age by Group Interactions**				
	Typically Developing by Psychosis Spectrum		Typically Developing by Other Psychopathologies	
Agranular	-.011	.696	.018	.497
Dysgranular	-.001	.957	.012	.646
Granular	-.027	.323	-.046	.092
**Gyrification**	**Statistics[a]**			
	$\mathbf{R_{partial}}$	**p**	$\mathbf{R_{partial}}$	**p**
**Main Effect of Age**				
Agranular	.167	<.001	--	--
Dysgranular	.167	<.001	--	--

Granular	.016	.562	--	--
**Age by Group Interactions**				
	Typically Developing by Psychosis Spectrum		Typically Developing by Other Psychopathologies	
Agranular	.005	.168	.001	.981
Dysgranular	-.017	.542	.029	.292
Granular	.021	.449	.086	.001
**Sulcal Depth**	**Statistics[a]**			
	**$R_{partial}$**	**p**	**$R_{partial}$**	**p**
**Main Effect of Age**				
Agranular	-.241	<.001	--	--
Dysgranular	-.349	<.001	--	--
Granular	-.361	<.001	--	--
**Age by Group Interactions**				
	Typically Developing by Psychosis Spectrum		Typically Developing by Other Psychopathologies	
Agranular	-.011	.679	.02	.452
Dysgranular	.011	.691	.013	.626
Granular	.025	.363	.012	.666

Table S7: Sex Effects: Psychosis Cohort

Volume	Statistics	
	$R_{partial}^{a}$	p
**Main Effect of Sex**		
Agranular	-.07	.119
Dysgranular	-.069	.127
Granular	-.041	.359
**Sex by Group Interaction**		
Agranular	-.007	.877
Dysgranular	-.029	.514
Granular	.027	.550
**Cortical Thickness**	**Statistics**	
	$R_{partial}^{a}$	p
**Main Effect of Sex**		
Agranular	-.097	.032
Dysgranular	-.073	.104
Granular	-.04	.374
**Sex by Group Interaction**		
Agranular	.037	.410
Dysgranular	.013	.767
Granular	.058	.198
**Gyrification**	**Statistics**	
	$R_{partial}^{a}$	p
**Main Effect of Sex**		
Agranular	-.045	.320
Dysgranular	.075	.095
Granular	.134	.003
**Sex by Group Interaction**		
Agranular	-.036	.427
Dysgranular	.001	.983

Granular	-.076	.091
**Sulcal Depth**	**Statistics**	
	**$R_{partial}$[a]**	**p**
**Main Effect of Sex**		
Agranular	.120	.008
Dysgranular	.067	.136
Granular	.003	.946
**Sex by Group Interaction**		
Agranular	-.012	.783
Dysgranular	.031	.498
Granular	.025	.584

Table S8: Sex effects: PNC Cohort

**Volume**	**Statistics[a]**			
	**$R_{partial}$**	**p**	**$R_{partial}$**	**p**
**Main Effect of Sex**				
Agranular	-.022	.422	--	--
Dysgranular	-.073	.007	--	--
Granular	.036	.181	--	--
**Sex by Group Interactions**				
	Typically Developing by Psychosis Spectrum		Typically Developing by Other Psychopathologies	
Agranular	-.041	.133	-.011	.698
Dysgranular	-.042	.125	-.012	.667
Granular	-.037	.171	-.029	.284
**Cortical Thickness**	**Statistics[a]**			
	**$R_{partial}$**	**p**	**$R_{partial}$**	**p**
**Main Effect of Sex**				
Agranular	-.037	.167	--	--
Dysgranular	-.117	<.001	--	--
Granular	-.024	.382	--	--
**Sex by Group Interactions**				
	Typically Developing by Psychosis Spectrum		Typically Developing by Other Psychopathologies	
Agranular	.019	.480	.009	.752
Dysgranular	.047	.085	.004	.885
Granular	.034	.215	.019	.481
**Gyrification**	**Statistics[a]**			
	**$R_{partial}$**	**p**	**$R_{partial}$**	**p**
**Main Effect of Sex**				
Agranular	-.089	.001	--	--
Dysgranular	.002	.932	--	--

Granular	.036	.190	--	--
**Sex by Group Interactions**				
	Typically Developing by Psychosis Spectrum		Typically Developing by Other Psychopathologies	
Agranular	-.046	.087	-.069	.011
Dysgranular	-.017	.520	.027	.323
Granular	-.03	.274	-.027	.316
**Sulcal Depth**	**Statistics[a]**			
	**$R_{partial}$**	**p**	**$R_{partial}$**	**p**
**Main Effect of Sex**				
Agranular	.007	.785	--	--
Dysgranular	.048	.079	--	--
Granular	-.004	.880	--	--
**Sex by Group Interactions**				
	Typically Developing by Psychosis Spectrum		Typically Developing by Other Psychopathologies	
Agranular	-.022	.416	-.019	.493
Dysgranular	-.037	.177	-.013	.626
Granular	-.026	.331	-.028	.300

Table S9: Correlations between CPZ-equivalence values and insula sub-region metrics

	**Statistics**	
	**r**	**p**
Agranular Volume	-.112	.210
Dysgranular Volume	-.017	.853
Granular Volume	-.013	.883
Agranular Thickness	-.045	.612
Dysgranular Thickness	.067	.453
Granular Thickness	.015	.871
Agranular Gyrification	-.051	.571
Dysgranular Gyrification	-.151	.09
Granular Gyrification	-.147	.098
Agranular Sulcal Depth	-.026	.771
Dysgranular Sulcal Depth	-.065	.466
Granular Sulcal Depth	-.044	.62

Table S10: Demographics of matched PNC cohorts

	**Typically Developing** N=126	**Psychosis Spectrum** N=143	**Statistic**
Age	15.02 (2.68)	15.04 (2.48)	$F(1,267)=.004$, $p=.947$
Gender (Male/Female)	65/61	71/72	$X^2=.11$, $p=.136$
Race (White/Non-White)	58/68	52/91	$X^2=.75$, $p=.807$
Cognitive Ability	0.10 (0.46)	-0.06 (.55)	$F(1,265)=4.18$, $p=.042$

Figure S1: Unthresholded voxel-wise and vertex-based results

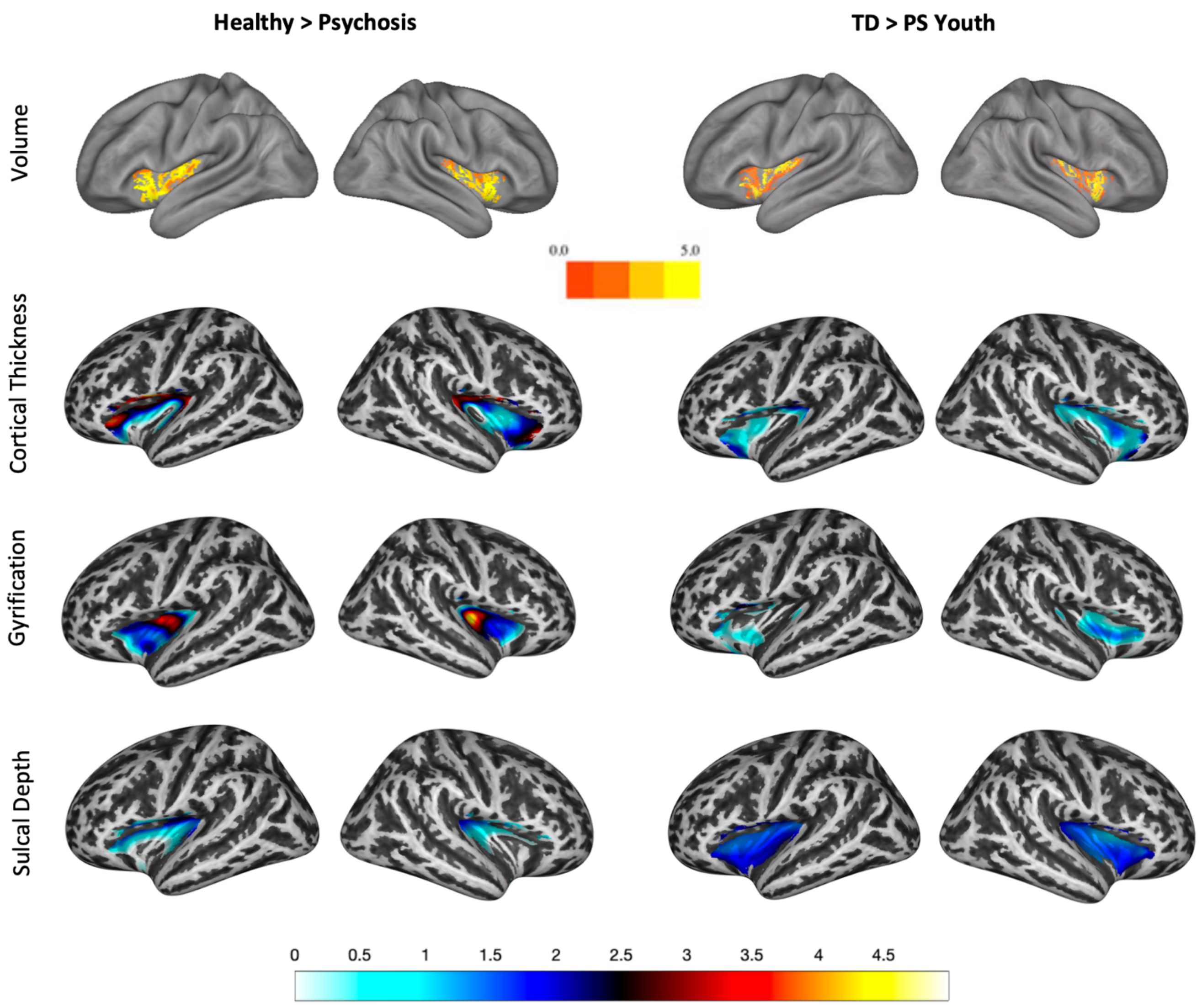

Contrast of across the whole insula (p<.99) to demonstrate overall pattern of volume differences between groups. Controlling for age, gender, TIV, topography defects (surface-based morphometry), study (psychosis cohort).

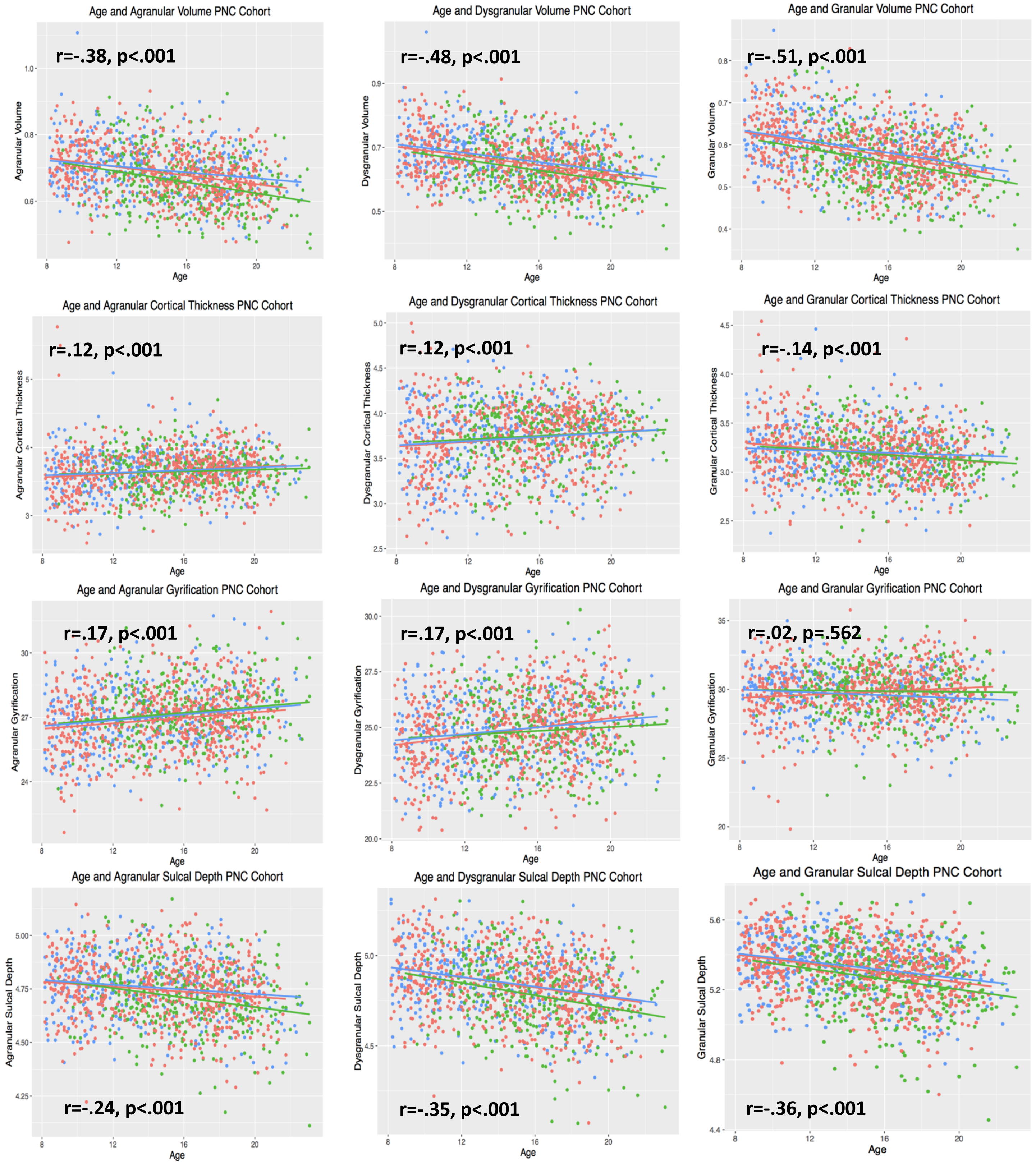
Age and Agranular Volume PNC Cohort
r=-.38, p<.001
Agranular Volume
Age
Age and Dysgranular Volume PNC Cohort
r=-.48, p<.001
Dysgranular Volume
Age
Age and Granular Volume PNC Cohort
r=-.51, p<.001
Granular Volume
Age
Age and Agranular Cortical Thickness PNC Cohort
r=.12, p<.001
Agranular Cortical Thickness
Age
Age and Dysgranular Cortical Thickness PNC Cohort
r=.12, p<.001
Dysgranular Cortical Thickness
Age
Age and Granular Cortical Thickness PNC Cohort
r=-.14, p<.001
Granular Cortical Thickness
Age
Age and Agranular Gyrification PNC Cohort
r=.17, p<.001
Agranular Gyrification
Age
Age and Dysgranular Gyrification PNC Cohort
r=.17, p<.001
Dysgranular Gyrification
Age
Age and Granular Gyrification PNC Cohort
r=.02, p=.562
Granular Gyrification
Age
Age and Agranular Sulcal Depth PNC Cohort
r=-.24, p<.001
Agranular Sulcal Depth
Age
Age and Dysgranular Sulcal Depth PNC Cohort
r=-.35, p<.001
Dysgranular Sulcal Depth
Age
Age and Granular Sulcal Depth PNC Cohort
r=-.36, p<.001
Granular Sulcal Depth
Age
Dx
OP
PS
TD

Figure S2

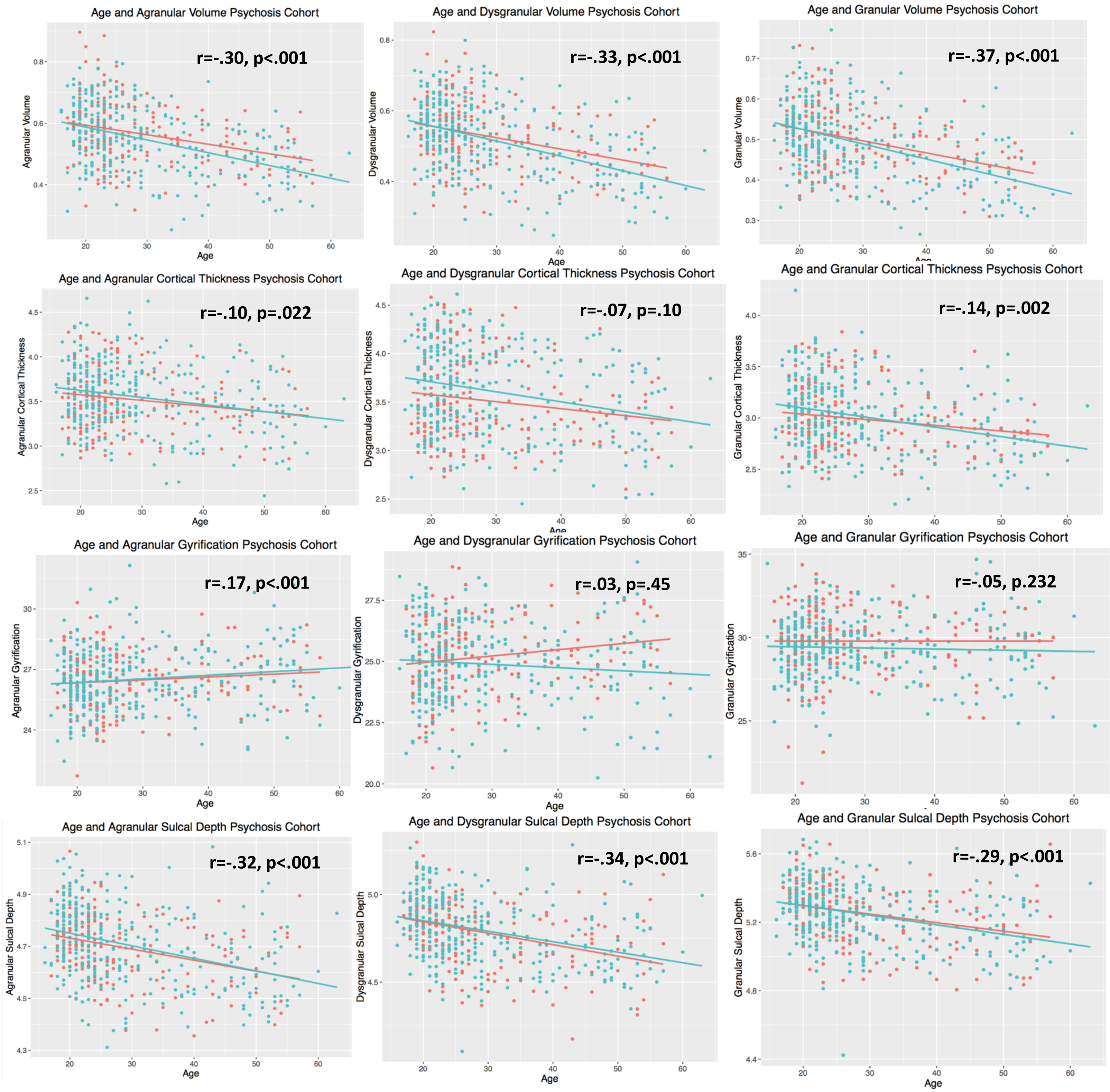
Age and Agranular Volume Psychosis Cohort
r=-.30, p<.001
Agranular Volume
Age
Age and Dysgranular Volume Psychosis Cohort
r=-.33, p<.001
Dysgranular Volume
Age
Age and Granular Volume Psychosis Cohort
r=-.37, p<.001
Granular Volume
Age and Agranular Cortical Thickness Psychosis Cohort
r=-.10, p=.022
Agranular Cortical Thickness
Age
Age and Dysgranular Cortical Thickness Psychosis Cohort
r=-.07, p=.10
Dysgranular Cortical Thickness
Age and Granular Cortical Thickness Psychosis Cohort
r=-.14, p=.002
Granular Cortical Thickness
Age and Agranular Gyrification Psychosis Cohort
r=.17, p<.001
Agranular Gyrification
Age
Age and Dysgranular Gyrification Psychosis Cohort
r=.03, p=.45
Dysgranular Gyrification
Age
Age and Granular Gyrification Psychosis Cohort
r=-.05, p.232
Granular Gyrification
Age and Agranular Sulcal Depth Psychosis Cohort
r=-.32, p<.001
Agranular Sulcal Depth
Age
Age and Dysgranular Sulcal Depth Psychosis Cohort
r=-.34, p<.001
Dysgranular Sulcal Depth
Age
Age and Granular Sulcal Depth Psychosis Cohort
r=-.29, p<.001
Granular Sulcal Depth
Age

Figure S3

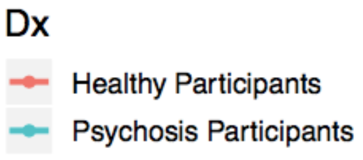
Dx
Healthy Participants
Psychosis Participants
